# On the Thermal Conductivity and Local Lattice Dynamical Properties of NASICON Solid Electrolytes

**DOI:** 10.1021/jacs.4c12034

**Published:** 2024-11-13

**Authors:** Thorben Böger, Tim Bernges, Matthias T. Agne, Pieremanuele Canepa, Frank Tietz, Wolfgang G. Zeier

**Affiliations:** a Institute of Inorganic and Analytical Chemistry, University of Münster, Münster D-48149, Germany; b International Graduate School for Battery Chemistry, Characterization, Analysis, Recycling and Application (BACCARA), University of Münster, Münster D-48149, Germany; c Department of Chemistry and Biochemistry, University of Oregon, Eugene, Oregon 97403, United States of America; d Department of Materials Science and Engineering, National University of Singapore, 117575Singapore; e Department of Chemical and Biomolecular Engineering, National University of Singapore, 117585Singapore; f Department of Electrical & Computer Engineering, University of Houston, Houston, Texas 77204, United States of America; g Institute of Energy Materials and Devices (IMD-2), Forschungszentrum Jülich, Jülich D-52425, Germany; h Institute of Energy Materials and Devices (IMD), IMD-4: Helmholtz-Institut Münster, Forschungszentrum Jülich, Münster 48149, Fed. Rep. Germany

## Abstract

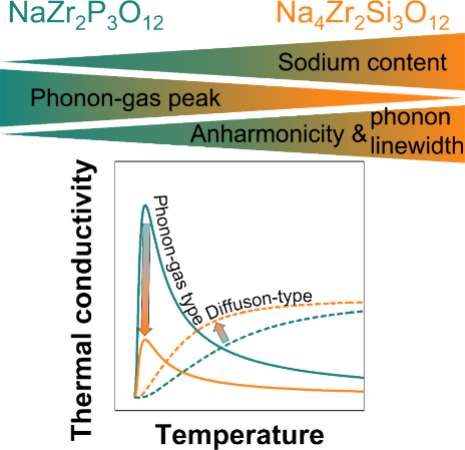

The recent development of solid-state batteries brings them closer to commercialization and raises the need for heat management. The NASICON material class (Na_1+*x*_Zr_2_P_*x*_Si_3–*x*_O_12_ with 0 ≤ *x* ≤ 3) is one of the most promising families of solid electrolytes for sodium solid-state batteries. While extensive research has been conducted to improve the ionic conductivity of this material class, knowledge of thermal conductivity is scarce. At the same time, the material’s ability to dissipate heat is expected to play a pivotal role in determining efficiency and safety, both on a battery pack and local component level. Dissipation of heat, which was, for instance, generated during battery operation, is important to keep the battery at its optimal operating temperature and avoid accelerated degradation of battery materials at interfaces. In this study, the thermal conductivity of NaZr_2_P_3_O_12_ and Na_4_Zr_2_Si_3_O_12_ is investigated in a wide temperature range from 2 to 773 K accompanied by in-depth lattice dynamical characterizations to understand underlying mechanisms and the striking difference in their low-temperature thermal conductivity. Consistently low thermal conductivities are observed, which can be explained by the strong suppression of propagating phonon transport through the structural complexity and the intrinsic anharmonicity of NASICONs. The associated low-frequency sodium ion vibrations lead to the emergence of local random-walk heat transport contributions via so-called diffusons. In addition, the importance of lattice dynamics in the discussion of ionic transport as well as the relevance of bonding characteristics typical for mobile ions on thermal transport, is highlighted.

## Introduction

Solid-state batteries use ion-conducting solids instead of liquid electrolytes and have been extensively studied in recent years.^1,2^ Especially limited lithium resources and a priority on cost-effectiveness make solid-state sodium batteries a promising option.^1,2^ Various categories of solid-state sodium-ion conductors are under examination, including the Na_3_PS_4_ family,^3^ borate-hydrides,^4^ the newly found oxyhalides,^5^ Na-β/β″-alumina,^6^ and the Na_1+*x*_Zr_2_P_*x*_Si_3–*x*_O_12_ (0 ≤ *x* ≤ 3) substitution series.^7^ The latter demonstrates superior thermal, chemical, and electrochemical stability, reducing the risk of thermal runaways, e.g., at the electrode/electrolyte interfaces.^8^ First reported by Hong^9^ and Goodenough et al.,^10^ these compounds are best known under the name Na^+^ superionic conductors (NASICONs) given their high ionic conductivity. The ionic conductivity of the series is the highest for *x* ≈ 2.4, with the endmembers, NaZr_2_P_3_O_12_ and Na_4_Zr_2_Si_3_O_12_, themselves being rather poor ionic conductors.^11^ By substitution of zirconium with other transition metals and even phosphorus and silicon with heavier homologues, a large variety of derived compounds can be obtained and the ionic conductivity can be increased to higher values.^7,12^

Typically, the focus in these materials is on maximizing sodium-ion conduction and its relation to structural features.^13−15^ To date, only a few studies aim to understand thermodynamic properties,^16−19^ such as heat capacity, or enthalpy and entropy of formation, or even the lattice dynamics^20,21^ in these materials. Recently, Morgan et al.^20^ and Zhen et al.^21^ employed *ab initio* phonon calculations for NaZr_2_P_3_O_12_ to improve the accuracy of chemical shift calculations for nuclear magnetic resonance and understand thermal expansion in NaZr_2_P_3_O_12_. Morgan et al.^20^ further used lattice dynamics calculations within the harmonic approximation to report anisotropic thermal displacement parameters and highlighted the importance of anharmonicity in this system. Zhen et al.^21^ reported phonon band structures and phonon density of states (DOS) and utilized the quasi-harmonic approximation to derive Grüneisen parameters. Although anharmonicity and phonon band structures are the basis for thermal transport in materials, not much is known about the thermal conductivity of these ion conductors.

When moving to large-scale applications, the thermal conductivity, which is dominated by the material’s lattice dynamics in electronic insulators, is of great importance.^22^ Hence, thermal conductivity characterizes the ability of solid electrolytes to dissipate heat generated during cycling.^23^ Good heat dissipation allows the heat to be removed faster, resulting in a lower equilibrium temperature of the battery (pack). High temperatures due to slow heat dissipation can drive the system out of its stable or at least optimal operating temperature. Therefore, ensuring all battery components are inside an optimal temperature window is indispensable to optimize battery performance and lifetime and to avoid device failure by thermal runaway. These considerations can be safeguarded by simulations predicting the temperature distribution inside a solid-state battery cell during cycling.^24,25^ As simulations rely on accurate experimental input parameters, comprehensive experimental knowledge about the thermal properties of all materials and a fundamental understanding of how heat distributes within a cell are required to evaluate solid-state battery safety.

Temperature gradients drive macroscopic thermal transport in the materials. At a microscopic scale, temperature differences translate to an inhomogeneous occupation of vibrational states, which thermodynamics seeks to balance.^26^ This balancing occurs by a net diffusion of heat-carrying phonons from the warmer to the colder region, which can macroscopically be observed as thermal conduction. The so-called phonon-gas model has traditionally been used in crystalline materials to describe this balancing rate.^27^ In this model, lattice vibrations, quantified as quasiparticles called phonons, carry thermal energy proportional to their heat capacity, mean free path length, and velocity. These phonons propagate through the crystal like atoms in an ideal gas until a scattering event occurs.^27,28^ In amorphous materials, the lack of long-range atomic order results in very small mean free path lengths that are in the order of interatomic distances and concurrently, very low thermal conductivities are observed.^29,30^ Thermal conductivities can often fall below the lower limit explained by the phonon-gas model, suggesting that the mean free path lengths of the phonons are below interatomic distances.^30,31^ Therefore, transport can no longer be treated solely by gas-like phonons.^30^ Instead, a second mechanism of phonon heat transport has been proposed, conducting heat via coupling of energetically degenerate vibrations.^32,33^ These nonpropagating phonon modes are called *diffusons*,^30^ which can be thought of as an atomic scale random-walk diffusion of thermal energy caused by a temperature gradient.^32^ Diffuson transport has also been reported in several crystalline materials with complex unit cells,^32^ leading to a large number of phonon modes, a sufficient degree of atomic disorder,^34,35^ or high levels of intrinsic anharmonicity.^34,36^ Computational methods have supported the hypothesis of diffuson-mediated thermal transport.^37,38^

**Figure 1:**
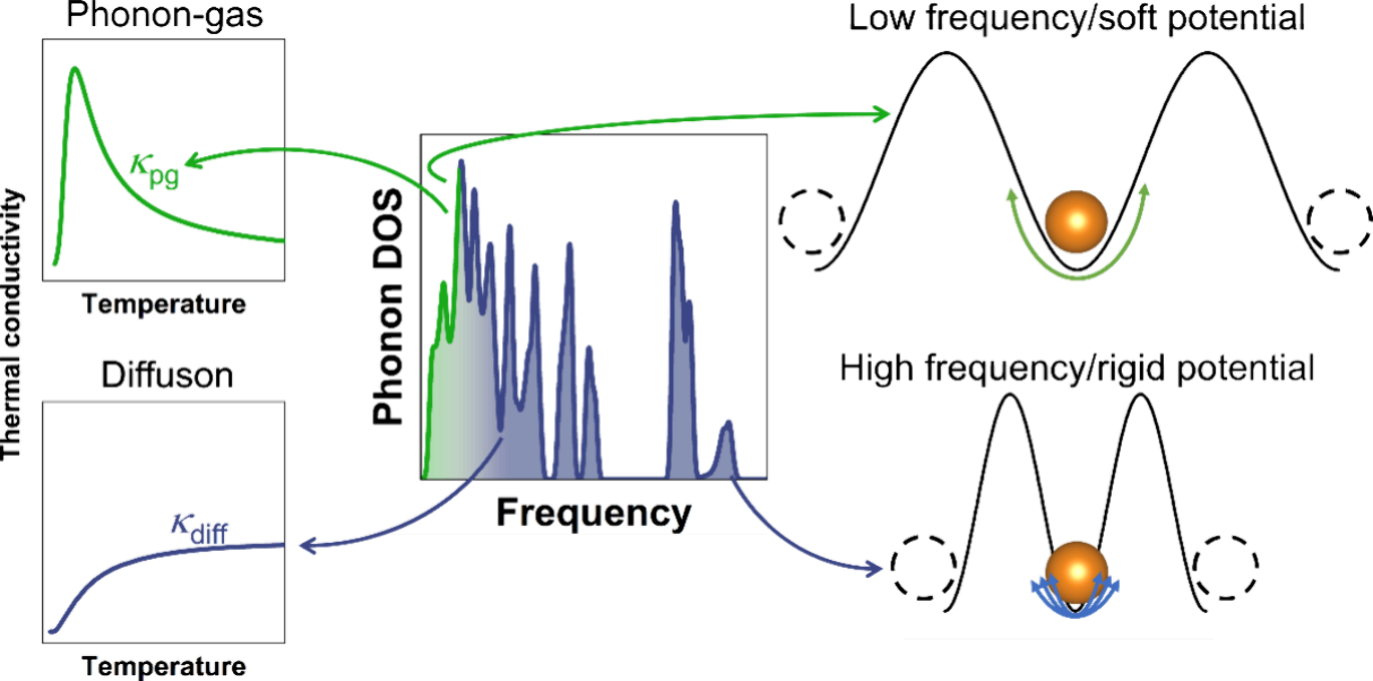
Schematic of the density of states (middle), indicating frequency ranges commonly associated with phonon-gas and diffuson character. Representation of typical thermal conductivity trends from phonon-gas and diffuson contributions (left). Low frequencies can be associated with softer potentials and larger vibrational amplitudes displacing the (mobile) ion further from its equilibrium position. In contrast, higher frequencies cause the ion to perform more periods of vibrations with smaller amplitudes (right).

Both the phonon-gas and the diffuson-type transport can occur in a material simultaneously, leading to theories accounting for thermal transport via both mechanisms in a parallel phonon-gas channel and a diffuson channel.^32,33,39^ As illustrated in Figure 1 (left), the thermal transport mechanisms can be differentiated empirically by the frequency range of their main contributions and their temperature dependence.^30,33^ Low energy (frequency) lattice vibrations, especially acoustic modes, typically contribute to the phonon-gas channel, whereas higher energy modes are rather diffuson-like.^30,35^ The Ioffe–Regel limit can be used as a rough guide to separate the phonon-gas and diffuson regime. It defines phonons whose lifetime τ is smaller than the inverse of their angular frequency ω (τ < 1/ω) as diffusons and is used in analytical models to divide the vibrational spectrum into phonon gas- and diffuson-like phonons.^30,35^ In more sophisticated models, like the two-channel model based on the Wigner transport equation developed by Simoncelli et al.,^33^ the thermal transport is calculated mode- and wave vector-dependent, resulting in a more gradual transition between phonon-gas-dominated and diffuson-dominated frequencies.

Commonly, thermal transport via propagating phonons is macroscopically manifested by a rapid increase in thermal conductivity at the lowest temperatures because of a rising heat capacity. A decrease proportional to the inverse of temperature occurs at higher temperatures due to enhanced phonon–phonon Umklapp scattering (Figure 1, upper left).^29^ The transition between both domains leads to a “phonon peak” in the thermal conductivity at low temperatures (usually below 50 K). Anharmonic interactions, necessary to describe phonon–phonon interactions and thermal conductivity, require a more sophisticated model than well-defined energy states for phonon modes. Anharmonicity gives rise to a distribution of phonon energies characterized by a Lorentzian profile centered around their harmonic frequencies. The breadth of this distribution is referred to as the phonon linewidth.^26^ Consequently, a certain phonon linewidth overlap of neighboring phonon modes emerges. Unlike the phonon-gas model, diffuson contributions increase with temperature because of increasing phonon occupation and overlap. At elevated temperatures, saturation of thermal conductivity occurs due to the inherent limit of fully overlapping modes, and diffusons are expected to conduct thermal energy at a constant rate (Figure 1, lower left).^32,33^

Motivated by the need to explore the thermal conductivity of solid electrolytes, this study examines the lattice dynamics of the endmembers of the NASICON series, namely, NaZr_2_P_3_O_12_ and Na_4_Zr_2_Si_3_O_12_, and elucidates the relevance of lattice dynamics for ionic and thermal transport in these materials. Although their ionic conductivities are low, understanding the dynamics in the NASICON endmembers can help comprehend the influence of dynamics on the entire series. The choice of the endmembers allows us to deconvolute potential outcomes of Na^+^ content, avoiding the complexities of sodium/vacancy and P/Si disorder.^11^ The thermal transport of NaZr_2_P_3_O_12_ and Na_4_Zr_2_Si_3_O_12_ is experimentally investigated in a wide temperature range from 2 to 773 K, revealing diffuson contributions to thermal transport in these solid electrolytes. The enhanced anharmonicity of the sodium sublattice in Na_4_Zr_2_Si_3_O_12_ compared to NaZr_2_P_3_O_12_ is assessed by mode-dependent Grüneisen parameters, rationalizing the differences in thermal conductivity observed in both compositions. Combining a spatial analysis of the average vibrational frequencies of sodium ions with the spectral breakdown of thermal conductivity underscores the importance of lattice dynamics in the discussion of the ionic transport properties (Figure 1, right). Additionally, this analysis shows that vibrations that are typically deemed important for ionic transport exhibit low frequencies and distinct anharmonicity. This work provides a conceptually different perspective on the interplay between thermal and ionic transport in solid electrolytes. A unique perspective on how energy landscapes shape characteristic frequencies in ionic conductors is provided. It demonstrates that the sublattice and site occupation of the mobile species, pivotal for ionic conductivity, also affects phonon linewidths and thermal transport in solid ionic conductors.

## Results and Discussion

### Crystal Structure and Thermal Expansion

**Figure 2:**
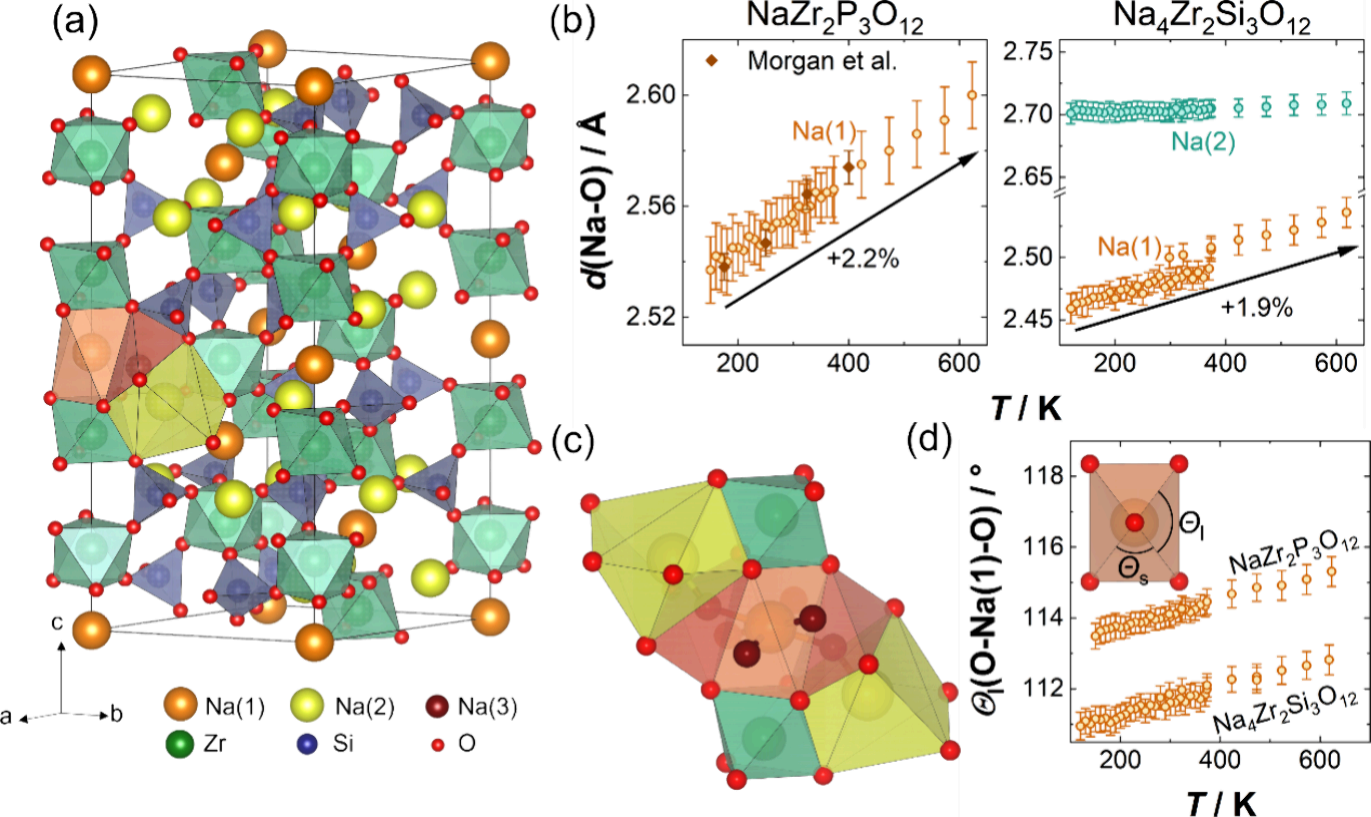
(a) Hexagonal representation of the unit cell of Na_4_Zr_2_Si_3_O_12_ with coordination environments and the connectivity of the Na^+^ sites. The only differences in the NaZr_2_P_3_O_12_ structure are the vacant Na(2) sites and PO_4_^3–^ instead of SiO_4_^4–^ tetrahedra. (b) Temperature dependence of the Na–O bond distances. For the Na(2) site in Na_4_Zr_2_Si_3_O_12_ the average bond distance is given, and the temperature dependence of all four individual bond lengths is shown in Figure S7. (c) Connectivity of the sodium polyhedra, visualizing that the diffusion path from the Na(1) to Na(2) site is bent. Only 2 out of the 6 adjacent Na(3) polyhedra around the central Na(1) antiprism are shown for visual clarity. The connectivity to all adjacent Na(2) and Na(3) sites can be best seen in Figure 8a–c. (d) Diverging bond angles within the Na(1)O_6_ polyhedra due to the rotation of ZrO_6_^8–^ and (P/Si)O_4_^3/4–^ polyhedra building up the rigid framework of the unit cell, leading to a distortion of the sodium ion cavity.

NaZr_2_P_3_O_12_ and Na_4_Zr_2_Si_3_O_12_ both crystallize in a trigonal lattice within the *R*3̅*c* space group, independent of temperature.^14^ In this structure, the Zr^4+^ ions are coordinated by oxygen in octahedrons that are corner-sharing with PO_4_^3–^ and SiO_4_^4–^ polyanions, respectively. Vice versa, each tetrahedron is connected to one octahedron at each corner.^7,40^ In NaZr_2_P_3_O_12,_ sodium ions only occupy the 6-fold, antiprismatic coordinated sodium site Na(1) (orange, Figure 2a). In addition to the occupation of the Na(1) position, an irregularly 8-fold coordinated sodium site Na(2) is occupied in Na_4_Zr_2_Si_3_O_12_ (yellow, Figure 2a). The irregular shape of the Na(2) polyhedron results in four distinct bond lengths. In both NaZr_2_P_3_O_12_ and Na_4_Zr_2_Si_3_O_12_, the respective sodium positions are fully occupied.

The diffusion pathway between the two sodium sites Na(1) and Na(2) involves an intermediate, 5-fold coordinated position (typically referred to as Na(3)) located between Na(1) and Na(2). The Na(1) antiprisms are elongated along the *c*-axis and linked to six adjacent Na(2) and Na(3) sites, whereas each Na(2) site is only neighbored by two Na(1) sites.^41,42^ While ion motion is expected to involve the intermediate Na(3) position, resulting in a bent diffusion pathway from the Na(1) to the Na(2) site (Figure 2c), this site is not permanently occupied in any of the endmembers. This fully ordered sodium sublattice causes both compounds to have low ionic conductivities (see Section S2).^14^ As the Na(2) site serves as an intermediate position in NaZr_2_P_3_O_12_, the path between two permanently occupied sites involves twice as many individual jumps than in Na_4_Zr_2_Si_3_O_12_, leading to an even lower ionic conductivity.

To ensure the successful synthesis of NaZr_2_P_3_O_12_ and Na_4_Zr_2_Si_3_O_12_ and to acquire detailed structural information, temperature-dependent X-ray diffraction and Rietveld refinements were performed (details in Section S3). Diffraction confirms the high phase purity of the materials (less than 1 wt % ZrO_2_) and agrees with the structural parameters of previous reports.^20,43^ The refined diffractograms and tabulated structural parameters at 150, 298, and 623 K are given in the Supporting Information (Section S3). Besides the structural evolution, these measurements allow for validation of the lattice dynamics calculations and provide information about the vibrational frequencies of the atoms.

With increasing temperature, anisotropic expansion of the unit cell can be observed for both compositions. The *c*-lattice parameter expands, while the *a*- and *b*-lattice parameters decrease (Figures S4 and S5), which seems caused by subtle correlated rotations of the ZrO_6_^8–^ and (P/Si)O_4_^3/4–^ polyhedra (Figure S6).^44^ The ZrO_6_^8–^ and (P/Si)O_4_^3/4–^ polyhedra are assumed to be rigid units. Despite the decrease in the crystallographic *a, b*-direction, the unit cell volumes increase linearly (Figure S4 and S5). Volumetric thermal expansion coefficients can be determined from the linear increase in the temperature. The volumetric thermal expansion coefficients for NaZr_2_P_3_O_12_ and Na_4_Zr_2_Si_3_O_12_ are 1.2(5)·10^–5^ K^–1^ and 2.2(2)·10^–5^ K^–1^, respectively, in good agreement with the literature.^45,46^

Analyzing the temperature dependence of the bond lengths reveals differences in their local expansion behavior. The bonds within the rigid framework composed of the (P/Si)O_4_^3/4–^ tetrahedra and ZrO_6_^8–^ octahedra do not change significantly across the temperature range investigated, confirming the assumption of rigid unit modes (Figures S4 and S5). In contrast, the Na–O bond length of the octahedrally coordinated Na(1) site exhibits significant thermal expansion of 5.04(11)·10^–5^ K^–1^ and 4.3(2)·10^–5^ K^–1^ in NaZr_2_P_3_O_12_ and Na_4_Zr_2_Si_3_O_12_, respectively (Figure 2b), corresponding to an increase of 2.2% and 1.9% between 150 and 600 K. Using an expansion coefficient of the bond length allows for direct comparisons with the dilatation of other distances in the unit cell. In agreement with the anisotropic thermal expansion of the unit cell, the expansion of the Na(1)-polyhedra is more pronounced in the crystallographic *c*-direction, leading to increased distortion of the coordination octahedra instead of a general volume expansion (Figure 2d). The additional 8-fold coordinated Na(2) position occupied in Na_4_Zr_2_Si_3_O_12_ is characterized by four distinct bond lengths, two decreasing and two expanding upon heating (Figure S7). The absolute values of their expansion coefficients are comparable to or even surpass those found for the Na(1)–O bonds. However, the average bond length of Na(2)–O remains constant within the experimental uncertainty and over the entire temperature range (Figure 2b). Imagining the ZrO_6_^8–^ and (P/Si)O_4_^3/4–^ polyhedra as rigid unit modes, any changes in their arrangements will deform the softer sodium polyhedra. Here, a “softer” polyhedron refers to lower bond strength, indicated by lower charges, longer bonds, and lower average vibrational frequencies (Figure 4a).

**Figure 3:**
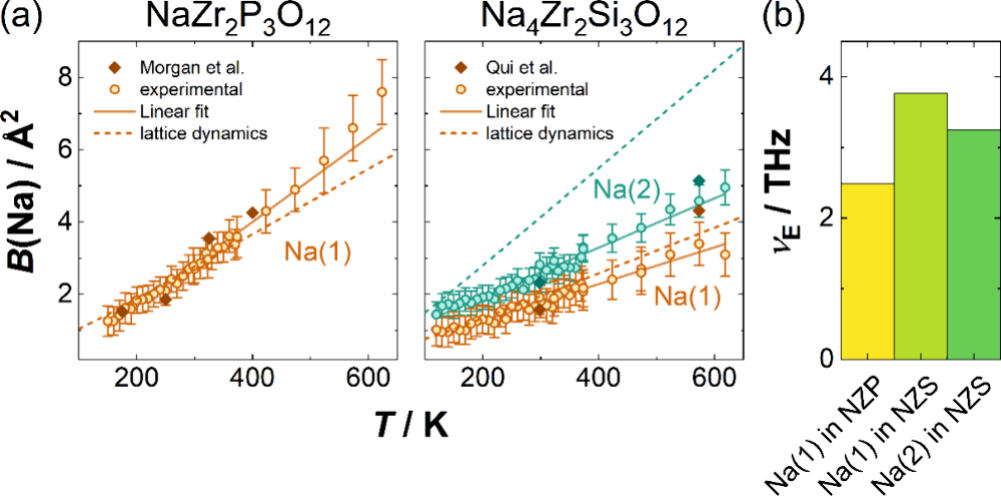
(a) Isotropic thermal displacement parameters from lattice dynamics calculations (dashed lines), values obtained from Rietveld refinement (symbols), and their respective linear fit (solid line). Diamond shapes correspond to values reported by Morgan et al.^20^ and Qui et al.,^43^ respectively. (b) Einstein frequencies of sodium ions on the Na(1) and Na(2) site in NaZr_2_P_3_O_12_ (NZP) and Na_4_Zr_2_Si_3_O_12_ (NZS), respectively, deduced from the temperature dependence of the isotropic thermal displacement parameters.

Alongside the volumetric expansion of the lattice and coordination polyhedra, a linear increase of the isotropic thermal displacement parameter of sodium on the Na(1) and Na(2) sites can be observed (Figure 3a). Former investigations and lattice dynamics calculations quantitatively confirm those values.^20,43^ From a lattice dynamics perspective, these high amplitudes of thermal vibration hint at loosely bound atoms with low force constants, as expected from the ion-conducting nature of the material. Low force constants of bonding lead to more deformable polyhedra, corroborating the significant thermal expansion coefficients of sodium–oxygen bonds discussed above. As the vibrational frequency of a bond scales with the square root of the force constants, the vibrational frequencies of sodium ions are expected to be comparably low. Vibrational frequencies can be approximated from the change of isotropic thermal displacements with temperature via Einstein frequencies (details in Section S4). The Einstein model treats each ion as vibrating as a harmonic oscillator independently of the others at a single frequency. With that, the vibrational frequency can be directly calculated from the experimental isotropic displacement parameter. The displacement parameters of the individual sodium sites differ, so a different frequency can be calculated for each distinct Na^+^ site. The steeper slope observed for the Na(1) site in NaZr_2_P_3_O_12_ yields a lower Einstein frequency of 2.49(4) THz compared to the sodium sites in Na_4_Zr_2_Si_3_O_12_ (3.77(7) THz and 3.25(5) THz for the Na(1) and Na(2) site, Figure 3b), suggesting lower force constants and weaker bonding interactions for NaZr_2_P_3_O_12_, caused by the longer Na(1)-O bond in NaZr_2_P_3_O_12_. This observation is supported by bond valence sum (BVS) analyses, which reveal a bond valence sum of 0.79 at room temperature for the Na(1) site in NaZr_2_P_3_O_12_, opposing to 0.91 in Na_4_Zr_2_Si_3_O_12_. The BVS can be seen as the effective number of electrons that an ion uses for bonding. Hence, sodium ions should approach a BVS of 1. Lower BVS generally indicates loosely bound species and, therefore, more mobile and weaker interacting ions. Considering the longer Na(1)–O bond distances and the more elongated coordination polyhedron in NaZr_2_P_3_O_12_ compared to Na_4_Zr_2_Si_3_O_12_ these findings align with the structural analysis. To analyze the bonding strength even further, the Crystal Orbital Hamilton Populations^47^ were calculated for both compounds (for computational details, see Section S1.4). The results corroborate the previous findings: Although there are no antibonding states below the Fermi level, the bonding energy of the sodium ions is much lower than those of zirconium, silicon, or phosphorus ions (see Figure S8).

**Figure 4:**
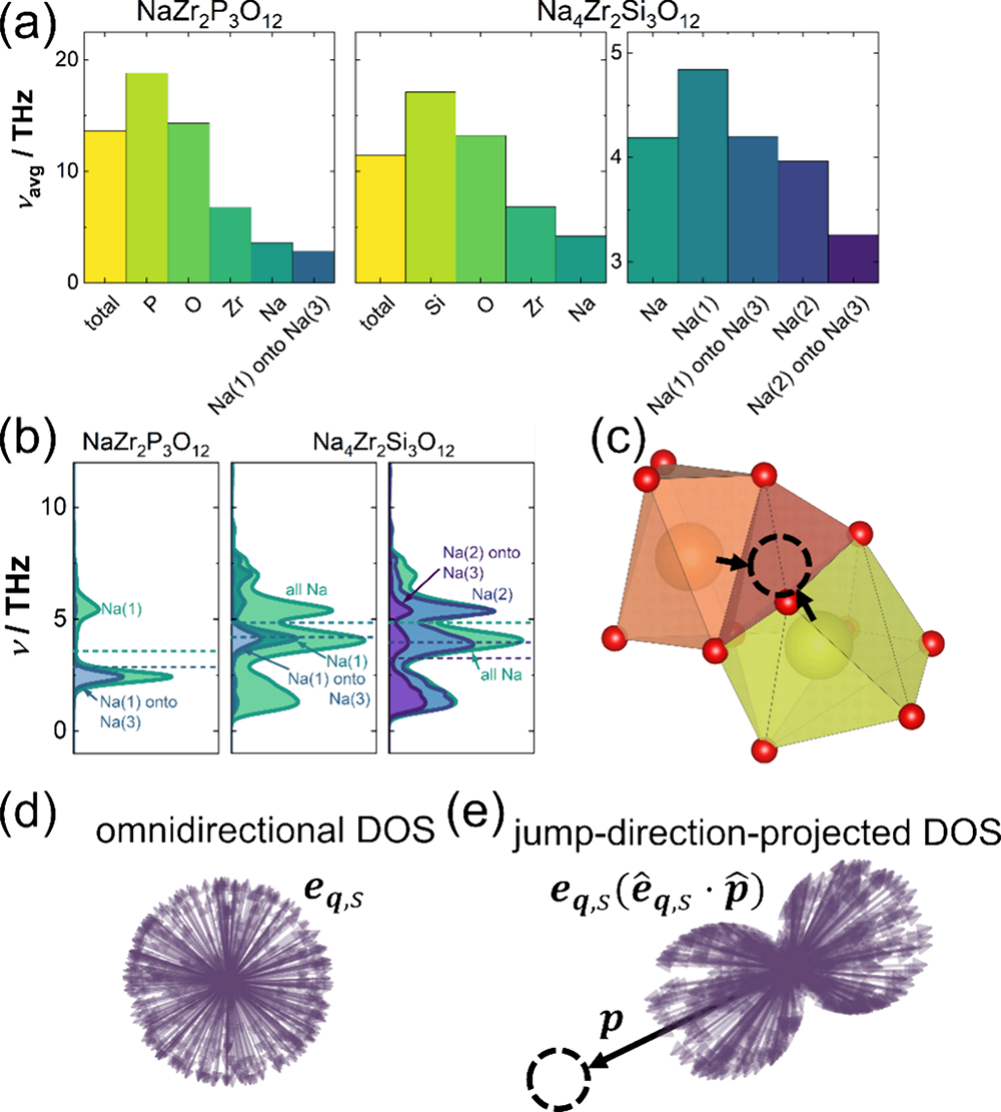
(a) Average frequencies of the total, atom-, site- and jump-direction-projected phonon DOS in NaZr_2_P_3_O_12_ in comparison to those found for Na_4_Zr_2_Si_3_O_12_. (b) Atom-, site- jump-direction-projected phonon DOS of sodium in NaZr_2_P_3_O_12_ and Na_4_Zr_2_Si_3_O_12_. In NaZr_2_P_3_O_12_, atom- and site-projected phonon DOS are identical as there is only one sodium site. (c) Connectivity of the Na^+^ polyhedra, visualizing the direction of projection for the jump-direction-projected phonon DOS (onto the Na(3) site). (d) Distribution of omnidirectional eigenvectors **e**_**q**,s_ and (e) distribution of eigenvectors projected onto the Na(3) site obtained by scaling the eigenvector by the dot product of the eigenvector and projection vector **p**. The hat notes that the respective vector is scaled to unity. The eigenvectors are a function of wave vector **q** and phonon branch index s.

### Lattice Dynamics

Given the inherent importance of lattice vibrations for thermal and ionic conductivities, *ab initio* lattice dynamics calculations were conducted for both NASICON compositions. From these calculations, e.g., the frequency distribution of phonon modes (i.e., the phonon density of states, phonon DOS) and the mode-resolved Grüneisen parameter as a measure for anharmonicity can be assessed to guide our understanding of transport in this material class. The dispersion relations in the form of a phonon band structure (Figure S9) reveal that most bands are almost dispersionless. The phonon DOS, integrated over the first Brillouin zone, contains information about the distribution of all of the phonon modes in the frequency space. The phonon DOS can be projected onto atomic species or distinct lattice sites to yield insights into their vibrational characteristics. For simplicity, the general vibrational characteristics of both compositions are not discussed in terms of the actual phonon DOS, but mainly in the form of average frequencies. However, the total phonon DOS and its projections are listed in the Supporting Information (Figure S10).

The overall shape of the phonon DOS does not change when phosphorus is replaced with silicon and sodium is added for charge compensation (Figure S10). Consequently, both compositions have similar average frequencies of the total phonon DOS, i.e., 13.6 and 11.4 THz for NaZr_2_P_3_O_12_ and Na_4_Zr_2_Si_3_O_12_, respectively. The consistent average frequency of the total phonon DOS can be explained by the similarities of the ZrO_6_–PO_4_ framework compared to the ZrO_6_–SiO_4_ framework. This structural similarity is reflected in the vibrational frequencies (Figure S10). As the ions in the framework are responsible for approximately 90% of all vibrations, the average vibrational frequencies between both compositions are also not altered significantly.

For further analyses of the vibrational spectrum, the phonon DOS can be projected onto each lattice site. Summing up contributions from all lattice sites of an atom yields the respective atom-projected phonon DOS. The similarity of the atom-projected phonon DOS of P and Si is also evident in their average vibrational frequencies with values of 18.8 and 17.1 THz, respectively (Figure 4a). We attribute these high-frequency vibrations to the rigidity of the PO_4_^3–^ and SiO_4_^4–^ frameworks, resulting in high force constants. All vibration modes involving Na^+^ ions are located at significantly lower frequencies, with average frequencies of 3.6 and 4.2 THz for both compositions (Figure 4a). Analyzing the two Na positions in Na_4_Zr_2_Si_3_O_12_ separately reveals a lower average frequency for the Na(2) site than the Na(1) site. Given the direct correlation of vibrational frequency and force constant, these low frequencies are a consequence of the weak bonding of sodium ions, resulting in high amplitudes of thermal vibration. Although still weakly bound, the bonding on the Na(1) position in Na_4_Zr_2_Si_3_O_12_ is expected to be stronger (higher average frequency) than that on the Na(2) site, which is confirmed by the structural analyses revealing shorter bond lengths and slightly lower absolute thermal expansion coefficients.

As Einstein frequencies measure only occupied phonon modes following the Bose–Einstein distribution, their frequencies are lower than the average frequencies obtained from lattice dynamics calculations. Weighing the phonon DOS by the phonon occupations of their frequencies, the average frequencies from lattice dynamics calculations at 300 K are reduced to 3.7 THz and 2.5 THz for the Na(1) and Na(2) site, respectively, which is in close agreement with the Einstein frequencies (Figure 3b and Figure S11).^48^

While the site-projected phonon DOS results in a complete spectrum of vibrations, taking displacements of the ions in all directions into account, it is also possible to project the phonon DOS into a specific spatial direction to probe the frequency of vibrations in that direction (Figure 4b–e). Projecting the phonon DOS in a specific direction can be regarded as only considering part of the phonon eigenvectors in that direction (Figure 4d,e). Again, the vibrational frequency scales directly with the force constant and is a proxy for the resistance ions experience when displaced in specific directions. Consequently, projecting the phonon DOS along the migration vector of the Na^+^ ion examines how different sodium vibrations contribute to the Na^+^ motion in the direction of the intermediate Na site (Na(3)), which in turn can quantify the restoring forces the ion faces during its diffusional motion (Figure 4b).

Considering Na_4_Zr_2_Si_3_O_12_ as both sodium sites, Na(1) and Na(2) are occupied, showing a significant reduction in average vibrational frequency from the site average to the jump-direction-projected average (4.8 to 4.2 THz and 4.0 to 3.3 THz for the Na(1) and Na(2) site, respectively) and suggesting that vibrations displacing the sodium ions into the jump direction possess frequencies even lower than the site average (Figure 4a,b). Previously, it was suggested that ions with lower average phonon frequencies are more likely to overcome the activation barrier for a jump to an adjacent site.^49^ At first, this seems counterintuitive, as the lower energy of low-frequency vibrations leads to a higher phonon occupation number that accumulates the energy of the activation barrier. However, the Bose–Einstein statistic indicates a significantly higher occupation number for low-frequency vibrations, which in turn lead to a higher probability of low-frequency vibrations to provide sufficient energy for the ion to overcome the activation barrier compared to vibrations of higher frequency. The low frequencies along the migration vector may, hence, be an essential factor in understanding ionic transport from a phonon perspective. This focus on low frequency contradicts conventional arguments derived from transition state theory, which posits that high attempt frequencies enhance ionic transport.^50^ However, this reasoning neglects that high-frequency vibrations are significantly less occupied.^49^ While this Perspective examines the influence of phonons on ionic transport, phonons and their phonon DOS are considered more often when discussing thermal transport than when discussing ionic transport.

### Thermal Conductivity in the Framework of Two-Channel Transport

**Figure 5:**
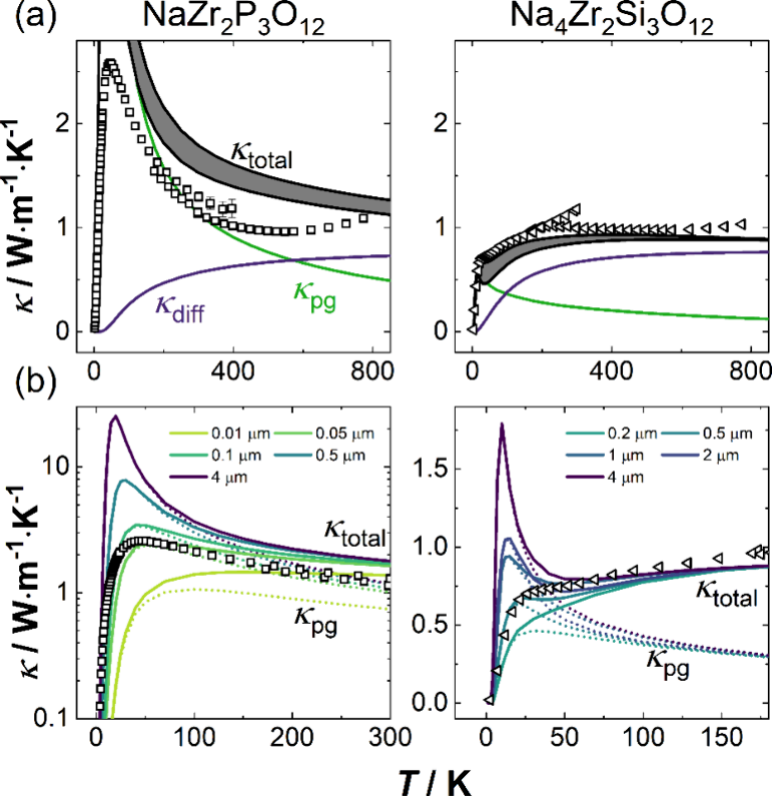
*Ab initio* modeled (lines) and experimental (symbols) thermal conductivities of NaZr_2_P_3_O_12_ and Na_4_Zr_2_Si_3_O_12_. (a) Side-to-side comparison of the materials with a crystallite size of 0.5 μm used to calculate the *ab initio* thermal conductivities. The anisotropic structure of both compounds results in anisotropic thermal conductivity. The lines shown for the total thermal conductivity represent the directions in space with the highest and lowest thermal conductivity. For visual clarity, phonon-gas and diffuson conductivities are averaged in all spatial directions. (b) Extension of panel (a) by different crystallite sizes to emphasize their influence (b) on the height of the phonon peak in both materials. Note that both panels are scaled differently due to a much more distinct phonon peak in NaZr_2_P_3_O_12_. The impact on the diffuson channel is negligible and therefore not shown.

Although the thermal conductivity is relatively insensitive to the overall shape of the vibrational spectrum, changes, especially at low frequencies, can alter the thermal conductivity’s features.^51^ Therefore, the thermal conductivity of NaZr_2_P_3_O_12_ and Na_4_Zr_2_Si_3_O_12_ is assessed experimentally in a broad temperature range from 2 to 773 K, which is necessary to identify the underlying transport mechanisms from the characteristic temperature dependencies. As NASICONs are electronic insulators, the contribution of the mobile electrons to the thermal conductivity is several orders of magnitude lower than their lattice thermal conductivity (for details, see Section S7). Both compositions exhibit similar thermal conductivities and temperature dependencies (Figure 5a) above approximately 350 K, but significant differences can be observed at low temperatures.

A phonon peak, characteristic of phonon-gas transport and the onset of dominant phonon–phonon scattering, is observed for NaZr_2_P_3_O_12_ at 45 K (Figure 5a left). With increasing temperature, the thermal conductivity shows a *T*^–1^-type decline, suggesting phonon scattering before saturating to constant values around 400 K. Generally, the high-temperature magnitudes are in agreement with previous reports by Rohde et al.^52^ for the solid solutions Na_2.7_Zr_2_P_1.3_Si_1.7_O_12_ and Na_3_Zr_2_PSi_2_O_12_. In contrast to NaZr_2_P_3_O_12_, no pronounced phonon peak is observed in Na_4_Zr_2_Si_3_O_12_ (Figure 5a, right). Instead, the initial increase in thermal conductivity via the phonon-gas channel causes a substantial increase in the total thermal conductivity up to 30 K followed by a less pronounced increase in thermal conductivity via diffusons.

A unified approach must be used to understand the temperature dependencies and the differences between both materials, considering phonon-gas and diffuson transport. Phonon-gas- and diffuson-type transport contributions are evaluated using an *ab initio* two-channel model proposed by Simoncelli et al.^33^ The model is based on calculations of third-order force constants that capture anharmonic interactions and can thereby predict phonon scattering (rates) and phonon linewidth broadening. Subsequently, third-order force constants are used to calculate the scattering of propagating phonons (phonon-gas channel) and the overlap and coupling of diffuson modes. This allows the calculation of the thermal conductivity contribution of every pair of two phonon branches, *s* and *s’*, at each temperature and *q*-point, instead of using a simple cutoff to distinguish between phonon gas- and diffuson-type conductivity. For *s* = *s’*, thermal transport is phonon gas-like; otherwise, for *s* ≠ *s’*, thermal transport occurs via the diffuson channel. Computational details are given in the Supporting Information (Section S1.4).

The total thermal conductivity predicted by the *ab initio* model describes the experimental results well, not requiring additional fourth-order force constants, which are necessary to describe the thermal conductivity in other materials.^53,54^ As the NASICON structure is anisotropic, the thermal conductivity is slightly different along the *c*-axis compared to the *ab*-plane. The range of thermal conductivity due to anisotropy is highlighted by the shaded area in Figure 5a. The total magnitude of the phonon peak is strongly related to the rate of defect scattering, e.g., grain boundaries (Figure 5b), making precise knowledge of the microstructure necessary. Scanning electron microscopy reveals similar microstructures in both compounds (see Section S8), with secondary particle sizes in the low micrometer range and below. However, these particle sizes only represent upper bounds for the crystallite sizes, but their actual size remains unknown. Additionally, the crystallite size within a sample is not monodisperse, as assumed by the phonon lifetime model used here, but will have some distribution.

Within these approximations, a crystallite size of 0.5 μm was estimated that fits the data of Na_4_Zr_2_Si_3_O_12_ best. The same analytical grain size was used for both compounds to avoid obfuscating the comparison between the two compounds and their analysis. However, in NaZr_2_P_3_O_12_ (Figure 5a left), the phonon peak at low temperatures is overestimated by the *ab initio* modeling using this crystallite size, and smaller crystallite sizes of ≈0.1 μm describe the phonon peak in NaZr_2_P_3_O_12_ more accurately. Figure 5b highlights the influence of varying crystallite sizes on the magnitude of the phonon peak magnitude.

Comparing the results for both NASICON compositions and distinguishing the contributions by phonon-gas-like and diffuson-like transport reveals that the significant difference is a stronger suppression of the phonon-gas contributions. This leads to differences in the temperature dependencies and an earlier (in temperature) increase and saturation of the diffuson contributions in Na_4_Zr_2_Si_3_O_12_. Given the similarity in heat capacity, speed of sound, phonon band structure, and sample treatment of both NASICON compositions, the stronger suppression of the phonon-gas channel seems to be driven by stronger phonon scattering in Na_4_Zr_2_Si_3_O_12_ caused by the additional sodium ions. There are no indications that other factors, such as strain, dislocations, or increased mass contrast, reduce the phonon peak in Na_4_Zr_2_Si_3_O_12_.

**Figure 6:**
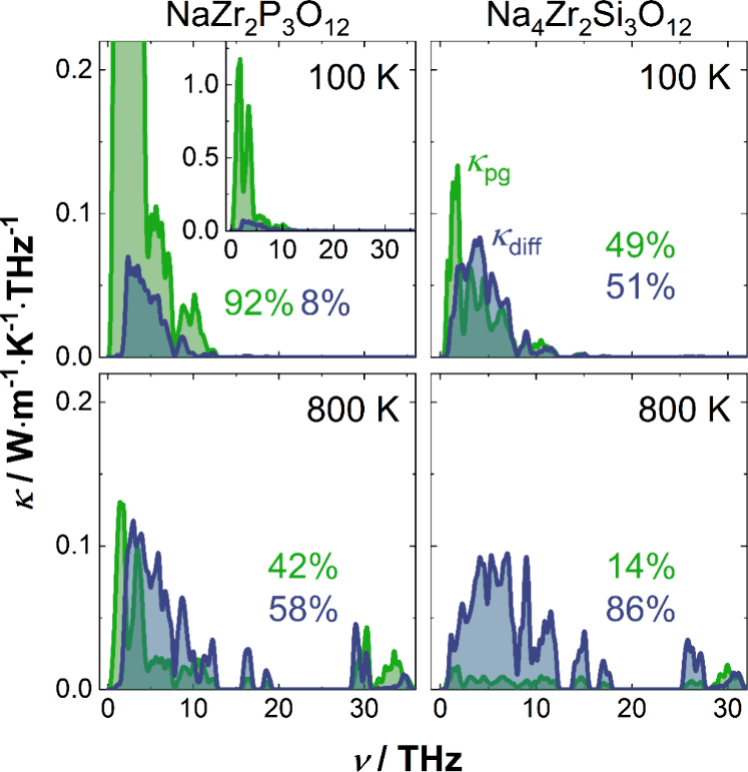
Spectral thermal conductivity of phonon-gas and diffuson channel, denoted as κ_pg_ and κ_diff_, respectively, at 100 (top panels) and 800 K (lower panels). Due to the high phonon peak at low temperatures in NaZr_2_P_3_O_12_, an inset is added to show the entire spectrum in the upper left panel. The percentages give the contribution of each channel to the total thermal conductivity.

**Figure 7:**
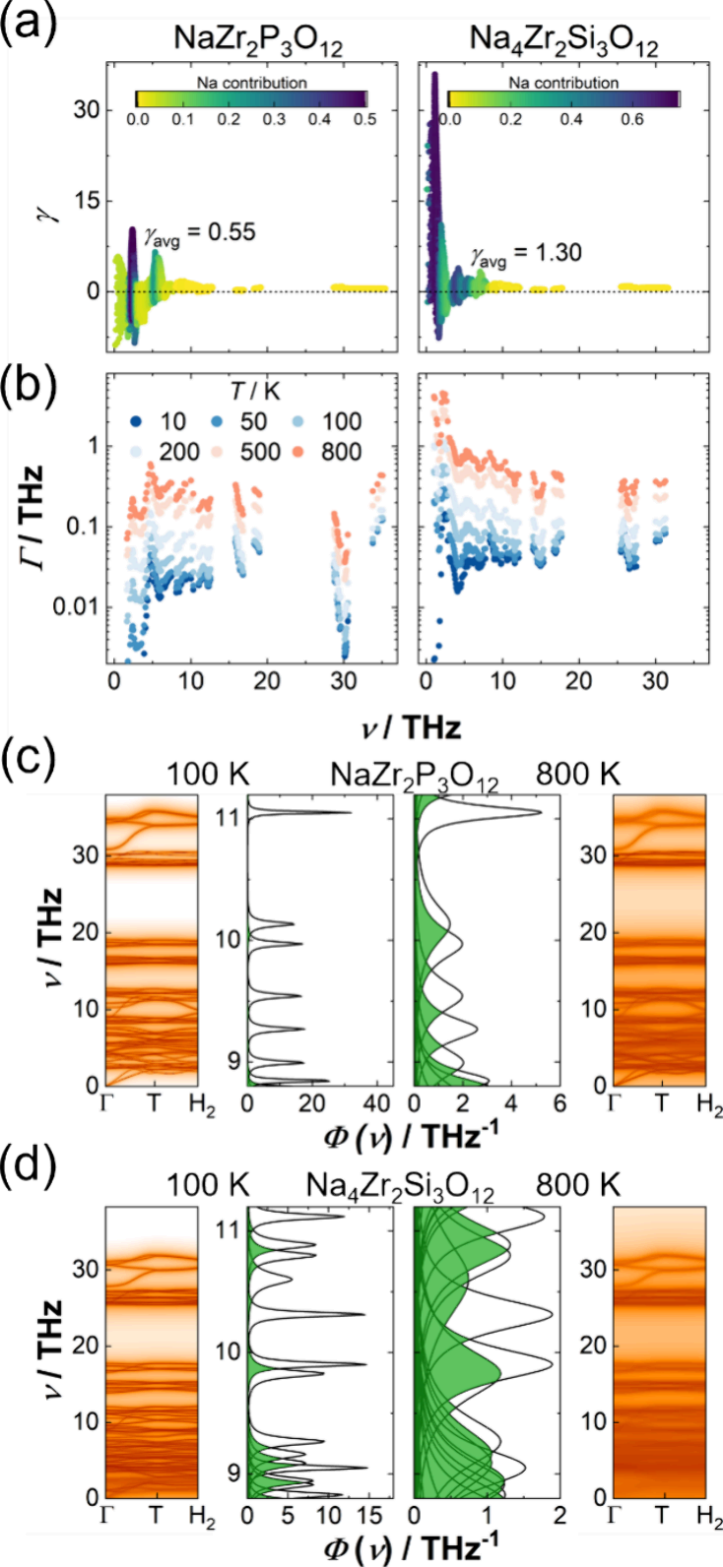
(a) Grüneisen parameter of NaZr_2_P_3_O_12_ (left panels) and Na_4_Zr_2_Si_3_O_12_ (right panels). The ratio of sodium partial phonon DOS to total phonon DOS, which is the contribution of sodium ions to vibrations at a given frequency, is color-coded. (b) Log-scaled phonon linewidths averaged over the Brillouin zone depicted at different temperatures. Both quantities exhibit highly anharmonic modes at low frequencies, especially for Na_4_Zr_2_Si_3_O_12_ at high temperatures. (c,d) Outer panels: Portion of phonon band structure at 100 and 800 K with color-coded phonon linewidths of (c) NaZr_2_P_3_O_12_ and (d) Na_4_Zr_2_Si_3_O_12_, respectively. Inner panels: Phonon spectral energy density of phonon bands in a selected frequency range, showing the effect of the phonon linewidth (broadening) on the phonon overlap, highlighted with green shading.

To understand this difference from a fundamental lattice dynamics perspective, the spectral distributions of the thermal conductivities of both channels (Figure 6), Grüneisen parameters, and phonon linewidths for both compositions have been evaluated (Figure 7). Phonon-gas contributions are found predominantly at low frequencies, mostly below 5 THz. In contrast, dominant diffuson contributions spread the entire frequency range (Figure 6), aligning with the two-channel thermal conductivity theory.^30^ At elevated temperatures, contributions to the thermal conductivity at higher frequencies arise (lower panels in Figure 6). This can be reasoned by the enhanced occupation of high-frequency phonon modes at higher temperatures. With increasing temperature, the phonon-gas contributions are diminished.

In NaZr_2_P_3_O_12_, substantial contributions from the phonon-gas channel are evident across all temperatures, whereas the phonon-gas channel is largely suppressed at high temperatures in Na_4_Zr_2_Si_3_O_12_ (compare lower and upper panels in Figure 6). In contrast to the phonon-gas channel, diffuson-like thermal transport is more pronounced at higher temperatures. In line with the earlier increase of the diffuson channel in Na_4_Zr_2_Si_3_O_12_, higher diffuson contributions are found compared to NaZr_2_P_3_O_12_ (compared to the left and right panels in Figure 6). The comparison of Grüneisen parameters γ (Figure 7a) highlights distinct differences between the compositions. Na_4_Zr_2_Si_3_O_12_ exhibits a significantly stronger peak of the Grüneisen parameter at lower frequencies, i.e., γ_max_ = 36.0, at 1.1 THz for Na_4_Zr_2_Si_3_O_12_ as compared to γ_max_ = 10.3, at 2.4 THz for NaZr_2_P_3_O_12_. Especially this peak at low frequencies, where phonon-gas contributions are the strongest, can be considered as the underlying reason the phonon-gas contributions in this frequency range are completely suppressed at 800 K in Na_4_Zr_2_Si_3_O_12_ leading to the observed temperature dependence of the phonon-gas channel (Figure 5a). The significantly smaller and more evenly spread Grüneisen parameter in NaZr_2_P_3_O_12_ allows for a higher phonon peak and relevant phonon-gas contributions across all temperatures.

**Figure 8:**
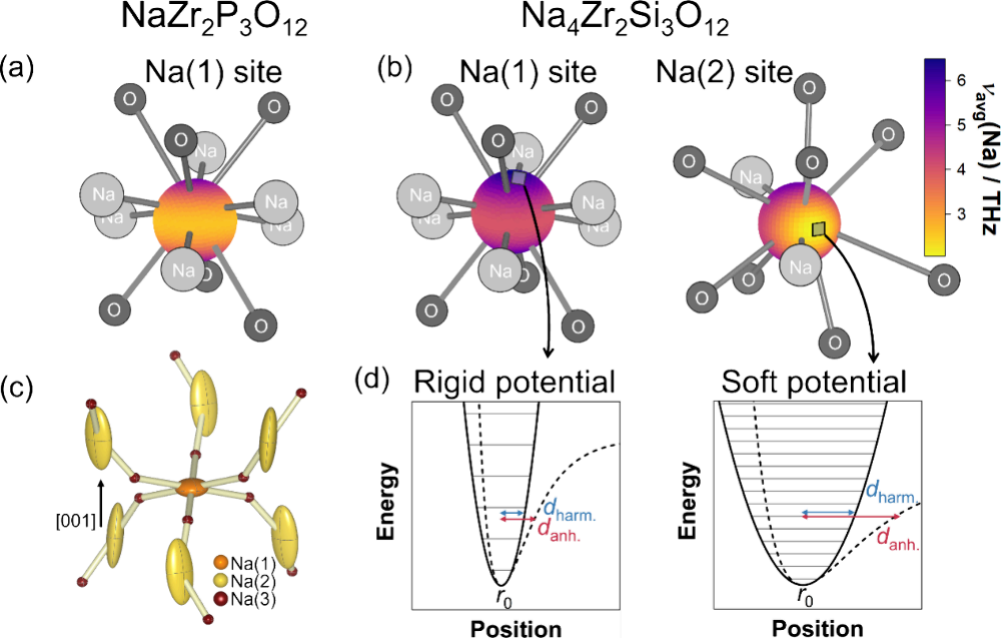
Local environment and vibration of the sodium sites: Spatial distributions of average site frequencies in NaZr_2_P_3_O_12_ (a) and Na_4_Zr_2_Si_3_O_12_ (b) when projected in a specific direction. The neighboring Na(3) and oxygen sites are shown for orientation. (c) Calculated anisotropic thermal displacement ellipsoids of the sodium ions in Na_4_Zr_2_Si_3_O_12_ at 300 K with 95% probability. The thermal displacement in the direction of diffusion is higher than average. Especially the displacement parameters of the Na(2) site are highly anisotropic. The Na(3) sites displayed in (a) to (c) are unoccupied and only added as a visual reference for the direction of the diffusion path. Their displacement parameters are arbitrarily chosen. (d) High average frequencies correspond to a more rigid potential well, smaller displacements (labeled d_harm._ and d_anh._), and less deviation of harmonic and anharmonic potential than lower frequencies. The eigenvalues of the harmonic oscillator, marked by the gray lines, have a larger spacing the more rigid the potential becomes.

The average Grüneisen parameters, which can be seen as a measure of the anharmonicity in the entire structure, are not extraordinarily high (γ = 1.51 for Na_4_Zr_2_Si_3_O_12_ and γ = 0.55 for NaZr_2_P_3_O_12_ at 300 K). Thus, the compounds are not particularly anharmonic, but the Na^+^ vibrations that dominate at frequencies with high Grüneisen parameters (color-coded in Figure 7a). Examining the sodium ion contribution to the phonon DOS per site reveals that the very high Grüneisen parameters at low frequency in Na_4_Zr_2_Si_3_O_12_ are associated with high contributions from ions on the Na(2) site, i.e., the sodium introduced by substitution of phosphorus with silicon (Figure S12). The distinct anharmonicity of the Na(2) site might be caused by its loose bonding (cf. Figure S7 and Figure 4b) compared to the Na(1) site and especially ions of the rigid framework, as indicated by its large thermal displacements (Figure 3). The Na(2) site is not occupied in NaZr_2_P_3_O_12_, which contributes to the lower anharmonicity observed in the phosphorus endmember. Considering that the high Grüneisen parameter is caused by the introduction of additional sodium ions to the Na(2) site, the strong reduction of the phonon-gas channel in Na_4_Zr_2_Si_3_O_12_ can be ultimately attributed to the introduction of additional sodium ions into the lattice too. The high Grüneisen parameters for sodium-ion vibrations are consistent with the low average frequencies and large amplitudes of the vibrations. Large amplitudes of vibration entailing large displacements from the equilibrium positions promote anharmonicity, as they cause more significant differences between harmonic and anharmonic bonding models (cf., Figure 8d).

While an increase in anharmonicity at low frequencies can explain the strong suppression of the phonon-gas channel, evaluating the phonon linewidth gives an explanation of why the diffuson contributions arise and govern the total thermal conductivity at lower temperatures in Na_4_Zr_2_Si_3_O_12_ than in NaZr_2_P_3_O_12_ (Figure 5a). In the harmonic approximation, the phonon bands are lines of zero width. However, considering anharmonicity leads to a finite width of the bands, known as phonon linewidth. Consequently, the frequency of each band becomes a probability distribution. The phonon linewidth directly influences this probability distribution of frequencies (called the phonon spectral energy density Φ), as it is the half-width at half-maximum of the distribution, which takes the form of a Lorentzian distribution:
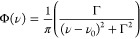
1where Γ is the phonon linewidth, ν the frequency, and ν_0_ the center frequency. Φ, Γ, and ν_0_ are all functions of wave vector ***q***, and phonon branch index *s*. As the phonon linewidths rise along with anharmonicity, just as the Grüneisen parameter, higher linewidth broadening is expected for Na_4_Zr_2_Si_3_O_12_. The linewidths in Na_4_Zr_2_Si_3_O_12_ are almost an order of magnitude higher, and with that, average linewidths of ≈0.1 THz are reached in NaZr_2_P_3_O_12_ only at 500 K, while these values are already reached at 200 K in Na_4_Zr_2_Si_3_O_12_ (Figure 7b). Despite these large linewidths, the scattering rate, which is directly related to the phonon linewidth, is approximately 1 order of magnitude lower than the phonon frequency. This in turn means that the phonon lifetime is still larger than the inverse of the angular frequency (τ > 1/ω), not passing the Ioffe–Regel limit. Increased linewidths directly facilitate the overlap of adjacent (in frequency) phonon modes (Figure 7c,d) and thereby directly increase the possibility of diffuson-type thermal transport.^33^ The broader the linewidths become, the greater the modes overlap, ultimately approaching the inherent limit of completely overlapping, marking an upper limit for the diffuson thermal conductivity and resulting in a saturating behavior. Given the lower temperatures for the same linewidth in Na_4_Zr_2_Si_3_O_12_, rise and saturation of the diffuson channel occur at lower temperatures too. Additionally, sizable primitive unit cells facilitate the overlap of phonon modes since more atoms in the primitive unit cell lead to more and, therefore, (in frequency) more closely spaced bands. While both endmembers already have comparably large unit cells, thus not requiring large linewidths for the phonon modes to overlap, Na_4_Zr_2_Si_3_O_12_ has slightly more bands (124) than NaZr_2_P_3_O_12_ (108), reducing the phonon linewidth needed even further.

Not only thermal but also ionic transport is an inherently anharmonic phenomenon, as the harmonic oscillator model fails to capture the saddle point between two lattice sites.^55^ Consequently, large thermal displacements, ion conduction, and anharmonicity are interrelated phenomena. This exact behavior can be found in Na_4_Zr_2_Si_3_O_12_. The additional Na(2) sites not only possess higher isotropic thermal displacement parameters than the Na(1) site but also their thermal displacement is highly anisotropic (Figure 8c), thereby amplifying the overall anharmonicity within the compound. The distinct anisotropic shape of the thermal displacement ellipsoids of the Na(2) site can be related to a distorted bonding environment with four different Na(2)–O bond lengths varying between 2.51 and 3.05 Å, resulting in spatially varying restoring forces, seen by the wider spread of average frequencies with the projection direction than on the Na(1) site (Figure 8b).

This anisotropy results from the vibrational spectrum being a function of the direction in which the spectrum is examined (cf. Figure 4 and 4e). From a phonon perspective, low frequencies are especially important to overcome the activation barrier for ion migration.^49^ Low phonon frequencies enhance ionic transport via two mechanisms. First, lower frequencies indicate lower force constants and therefore a softer potential, lowering the activation barrier. Here, Na_4_Zr_2_Si_3_O_12_ exhibits not only lower frequencies but also a lower activation barrier than NaZr_2_P_3_O_12_ as seen from impedance spectroscopy (Figure S2). Second, for a given activation energy, lower frequencies of the mobile ion enhance its probability to overcome the activation barrier.^49^ The spatial distribution of average vibrational frequencies (Figure 8b) allows us to visually evaluate ionic transport and diffusion path via lattice dynamics. Projecting the phonon DOS through the face of a coordination polyhedron will result in lower frequencies than a projection directly onto one of the coordinating ions, especially if the site behind the face is unoccupied. Therefore, the multitude of six adjacent Na(3) vacancies laying roughly in the *ab*-plane around the Na(1) site results in a band of low frequencies. As the Na(2) site is only neighbored by two Na(3) sites, two isolated areas of low frequency are observed.

These spatially variant projections of the phonon DOS are symmetric with respect to the sign of the projection vector, i.e., the projected phonon DOS, and thus, the average frequencies on opposing ends of the sphere are the same. Therefore, the area with the lowest average frequency does not align with both adjacent Na(3) sites, which form an angle of ≈140° with the Na(2) site. In contrast to the atom- and site-projected average frequencies, Grüneisen parameters, and phonon linewidths, these projections hold directional information. The average frequencies in the direction of ionic transport toward the adjacent intermediate Na(3) sites are extraordinarily low (e.g., 48% reduction for the Na(2) site in Na_4_Zr_2_Si_3_O_12_). Consequently, the dominant direction of the anisotropic thermal displacement parameters (Figure 8c) aligns well with the vector for ion migration. Low force constants correspond to a low average frequency and a high displacement from the equilibrium position (Figure 8d). This observation suggests that vibrations governing ionic transport exhibit predominantly low frequencies (low energies), and thus, low-frequency phonons should be mainly considered in the discussion of ionic transport, which corroborates the argument that low frequencies are most important to overcome activation barriers made from a phonon occupation perspective. Our data suggest that the number of low-energy attempt frequencies is very high because even at relatively low temperatures the low-energy modes contributed significantly to the Boltzmann partition function. The resulting rationale is therefore to prioritize the consideration of low-frequency phonons in the context of ionic transport dynamics. In this context, materials design strategies should aim for hierarchical bonding, promoting high frequencies perpendicular to and low frequencies in the direction of ionic transport due to their different bond strengths.

Additionally, pronounced anharmonicity linked with weakly bound and mobile ions is not limited to these NASICON compounds but should be applied to all solid electrolytes. As this anharmonicity is identified as the leading cause of low thermal conductivity, this might explain why generally low thermal conductivities are found in solid electrolytes.^56^ The difference in the temperature dependence of thermal conductivity between NaZr_2_P_3_O_12_ and Na_4_Zr_2_Si_3_O_12_ and the results of the lattice dynamics analysis can be summarized as follows:

1) Phonon-gas transport is suppressed significantly by large anharmonicities evoked by Na^+^ vibrations at low frequencies in Na_4_Zr_2_Si_3_O_12_.
2) At the same time, the increased anharmonicities facilitate mode coupling and diffuson transport.
3) Average frequencies in the direction of ionic transport are extraordinarily low. These vibrational modes are, therefore, of particular importance for ionic transport, and local structural modifications focusing on these modes may help in designing faster ionic conductors.

## Conclusions

In this study, the thermal conductivity and lattice dynamics of two NASICON solid electrolytes, NaZr_2_P_3_O_12_ and Na_4_Zr_2_Si_3_O_12_, were investigated through a combination of experiments and *ab initio* calculations. This investigation reveals that thermal transport at high temperatures is dominated by diffusons. Temperature-dependent X-ray diffraction experiments and lattice dynamics calculations reveal low average vibrational frequencies of sodium ions, corresponding to low force constants and weakly bound ions. By analyzing the spatial distribution of average phonon frequencies, particularly low frequencies in the direction of ionic transport become evident. These below-average frequencies demonstrate that vibrational modes with high anharmonicity and low frequencies, i.e., high phonon occupations, should be beneficial for ionic transport and not those with high frequencies as typically considered. The relevance of low-frequency vibrations marks an important point for the future design of ionic conductors, as one part of the design strategies often employed is the increase of attempt frequencies. The distinct anharmonicity of sodium vibrations causes a clear reduction of thermal transport via the phonon-gas channel and suppression of the phonon peak in Na_4_Zr_2_Si_3_O_12_. Examination of the phonon linewidths simultaneously revealed an increased overlap of adjacent modes promoting diffuson-type thermal transport at lower temperatures. With respect to the inherent anharmonicity of ion transport present in all solid electrolytes, this anharmonicity of the mobile ion sublattice appears to be a dominant factor in the low thermal conductivity observed for many solid electrolytes.

## Supporting Information Available

Experimental procedures, computational details, ionic conductivities, details on Rietveld refinements and structural analysis, theory of Einstein frequencies, Crystal Orbital Hamilton Populations, band structures, phonon density of states, electronic contribution to the thermal conductivity, scanning electron microscopy (PDF)
