# Supplementary material for: On the Thermal Conductivity and Local Lattice Dynamical Properties of NASICON Solid Electrolytes
Source: J Am Chem Soc. 2024 Nov 13;146(47):32678–88. doi: 10.1021/jacs.4c12034 (PMC11613629; doi:10.1021/jacs.4c12034)
Supplement: Supplementary file 1 — ja4c12034_si_001.pdf [file ja4c12034_si_001.pdf]

Supporting Information - On the thermal conductivity and local lattice dynamical properties of NASICON solid electrolytes

Thorben Böger[a,b], Tim Bernges[a], Matthias T. Agne[c], Pieremanuele Canepa[d,e,f], Frank Tietz[g,h], Wolfgang G. Zeier*[a,b,h]

[a]Institute of Inorganic and Analytical Chemistry, University of Münster, D-48149 Münster, Germany

[b]International Graduate School for Battery Chemistry, Characterization, Analysis, Recycling and Application (BACCARA), University of Münster, D-48149 Münster, Germany

[c]Department of Chemistry and Biochemistry, University of Oregon, Eugene, OR, 97403, United States of America

[d]Department of Materials Science and Engineering, National University of Singapore, 117575, Singapore

[e]Department of Chemical and Biomolecular Engineering, National University of Singapore, 117585, Singapore

[f]Department of Electrical & Computer Engineering, University of Houston, TX, 77204, United States of America

[g]Institute of Energy Materials and Devices (IMD-2), Forschungszentrum Jülich, D-52425 Jülich, Germany

[h]Institute of Energy Materials and Devices (IMD), IMD-4: Helmholtz-Institut Münster, Forschungszentrum Jülich, 48149 Münster, Germany.

Corresponding author emails: wzeier@uni-muenster.de

Section S1: Experimental Section

1.1 Synthesis

Powder of $NaZr_2P_3O_{12}$ was synthesized via solution-assisted solid state reaction based on a previous report[1] using NaCl (≥99.5%, Sigma-Aldrich), $ZrOCl_2 \cdot 8\ H_2O$ (98%, thermo scientific), and phosphoric acid (85 wt.%, Güssing GmbH). For densification of $NaZr_2P_3O_{12}$ 2 wt.% ZnO were added as sintering aid after calcination, the powder pressed into pellets, and subsequently sintered at 1000 °C for 24 h.[2]

For the synthesis of $Na_4Zr_2Si_3O_{12}$ $ZrO(NO_3)_2 \cdot 6\ H_2O$ (99%, Sigma-Aldrich), SiO_2 (99.5%, 10-20 nm particle size, Sigma-Aldrich), and Na_2SiO_3 (Sigma-Aldrich) were ground in an agate mortar for 15 minutes, pelletized, and subsequently fired at 1100 °C for 8 h. This procedure of grinding, pelletizing and firing was carried out four times in order to obtain low side-phase content and high degree of crystallinity. Densification to over 90% relative density was achieved by using 2 wt.% TiO_2 (99.9%, 32 nm APS Powder, thermo scientific) as sintering aid and heating to 1100 °C for 2 h.[3]

1.2 Thermal conductivity

Thermal conductivities above 173 K were obtained by thermal diffusivity measurements using a LFA 467 HyperFlash® setup by Netzsch. Either an InSb or a MCT (mercury cadmium telluride) detector was used for data acquisition for measurements above or below room temperature, respectively. The measurements were performed in inert atmosphere of pure nitrogen with a flow of 100 sccm. Samples were spray-coated with a graphite layer to enhance absorption and emission of infrared light. An improved version of the model proposed by Cape and Lehman was employed to calculate the thermal diffusivity.[5,6] Thermal conductivities were obtained by employing Equation 1, stating that the thermal conductivity is the product of thermal diffusivity D, density ρ, and specific isobaric heat capacity c_p.

$$\kappa = D \cdot \rho \cdot c_p \qquad (1)$$

The heat capacity is obtained from lattice dynamics calculation. As the isochoric, instead of isobaric, heat capacity is computed, these values have to be corrected by a dilatation term involving the bulk modulus B and the thermal expansion coefficient α (Equation 2). Due to the ultralow thermal expansion coefficient of NASICON materials, the dilatation term is negligible in first approximation and the isobaric heat capacity can be approximated very well by the isochoric heat capacity without additional terms.[7,8]

$$c_p(T) = c_V(T) + B\alpha^2 T \approx c_V(T) \tag{2}$$

Low temperature thermal conductivities (2 K to 400 K) were measured directly with a physical property measurement system (PPMS DynaCool manufactured by Quantum Design). Samples were measured in a disc geometry with a 3% temperature rise between hot and cold side. An emissivity value of 1.0 was assumed.

1.3 X-Ray Powder Diffraction

Temperature-dependent powder X-ray diffraction patterns were collected with a Stoe STADI P powder diffractometer in Debye-Scherrer geometry equipped with a Ge (111) monochromator and a Dectric Mythen 2X 1K detector using Mo $K_{\alpha 1}$ radiation (λ = 0.70930 Å). The diffractograms were measured in a 2θ range from 2° to 41° with a step size of 0.015°. In a range of 130 K to 370 K an Oxford Cryosystems Cryostream 1000 was utilized for adjusting the sample environment, from room temperature to 623 K a Stoe Insitu HT2 furnace was employed.

1.4 Density functional theory

All density functional theory calculations were carried out using the Vienna Ab initio Simulation Package (VASP)[9–11] utilizing projected-augmented-wave potentials[12]. The exchange correlation energy was calculated using the Perdew–Burke–Ernzerhof (PBE) functional within the generalized gradient approximation (GGA).[13] The PAW potentials used were Na_pv 19 Sep 2006 $2p^6 3s^1$, Zr_sv 04 Jan 2005 $4s^2 4p^6 4d^2 5s^2$, Si 05 Jan 2001 $3s^2 3p^2$, P 06 Sep 2000 $3s^2 3p^3$, and O 08 Apr 2002 $2s^2 2p^4$. Prior to any lattice dynamics calculations, the primitive unit cells of all materials were relaxed (details are given in Table S1). Subsequent lattice dynamics and thermal conductivity calculations were done using the phonopy[14,15] and phono3py[14,16] (details are given in Table S2 and Table S3, respectively). The thermal conductivity was computed solving the Wigner transport equation.[17,18] Grüneisen parameters were obtained from the quasi-harmonic approximation by additional lattice dynamics calculations on cells

whose volumes have been increased or decreased by 1%, respectively. The average Grüneisen parameter were calculated by averaging the mode-dependent Grüneisen parameter weight by their heat capacity:

$$\gamma_{\mathrm{avg}}(T) = \frac{\sum_{\boldsymbol{q},s} c_{V_{\boldsymbol{q},s}} \cdot \gamma_{\boldsymbol{q},s}}{c_V}$$
$$c_{V_{\boldsymbol{q},s}} = \frac{k_{\mathrm{B}}}{h} \sum_{\boldsymbol{q},s} \frac{x^2 \cdot \mathrm{e}^x}{(\mathrm{e}^x - 1)^2} \text{ with } x = \frac{h\nu_{\boldsymbol{q},s}}{k_{\mathrm{B}} T} \quad (3)$$

with γ denoting the Grüneisen parameter, c_V the isochoric heat capacity, T the thermodynamic temperature, k_B and h the Boltzmann and Planck constant, $\boldsymbol{q}$ the wavevector and s the phonon branch index.

While harmonic and quasi-harmonic lattice dynamics in phonopy utilize 2nd order force constants in combination with a non-analytical term correction[19,20], the calculation of the thermal conductivity with the Wigner transport equation in phono3py employ 3rd order force constants. More details on the phonopy post-processing can be found in Section S6.

Table S1. Computational parameters used for the structural relaxation of $NaZr_2P_3O_{12}$ and $Na_4Zr_2Si_3O_{12}$.

Computational parameter	***Value***
Energy cutoff / eV	600
k-points mesh	*Γ*-centered 7×7×7 (corresponding to a density > 10 Å)
Total energy convergence criterion / eV	10^{-8} (corresponding to < $3 \cdot 10^{-10}$ eV/atom)
Force convergence criterion / eV·Å^{-1}	10^{-3}

Table S2. Computational parameters used for phonopy calculations on $NaZr_2P_3O_{12}$ and $Na_4Zr_2Si_3O_{12}$.

Computational parameter	***Value***
Supercell size	2×2×2 (containing 288 and 336 atoms, respectively)
Energy cutoff / eV	600

Total energy convergence criterion / eV	10^{-8}
k-points mesh	3×3×3
Displacement distance / Å	0.01
q-mesh	20×20×20

Table S3. Computational parameters used for phono3py calculations on $NaZr_2P_3O_{12}$ and $Na_4Zr_2Si_3O_{12}$.

Computational parameter	***Value***
Supercell size	1×1×1 (containing 108 and 124 atoms, respectively)
Energy cutoff / eV	600
Total energy convergence criterion / eV	10^{-8}
k-points mesh	3×3×1
Displacement distance / Å	0.03
Cutoff pair-distance / Å	8.0
q-mesh	9×9×9

For the calculations of the Crystal Orbital Hamilton Populations[21] the LOBSTER package was used.[22] The analysis of the LOBSTER runs was done using the LobsterPy[23] package. The parameters used for the VASP calculations were the same as employed for the structural relaxation.

1.5 Impedance spectroscopy

Potentiostatic impedance spectroscopy was used to determine ionic conductivities using an Alpha-A impedance analyzer (Novocontrol Technologies). To minimize the grain boundary contributions to the impedance response, freestanding, sintered pellets were used for impedance spectroscopy. Electrical contact was ensured using Au electrodes, sputter-coated on each side, and Al current collectors. Pellets were sealed in pouch bags. Impedance spectroscopy was conducted in a temperature range of 153 K to 333 K. Each temperature was held for 1 h after meeting the stability criteria

of < 0.5 K deviation of the set temperature and a temperature change of < 0.5 K·min^{-1} before measuring impedance spectra to ensure a thermal equilibration. Spectra were recorded with sinusoidal excitation voltage with an amplitude of 35 mV in a frequency range of 10 mHz to 10 MHz.

Section S2: Ionic conductivity

Impedance responses of both compounds, $NaZr_2P_3O_{12}$ and $Na_4Zr_2Si_3O_{12}$, show two semicircles, i.e., processes in the Nyquist plot (Figure S1), and tail due to the blocking behavior of the gold electrodes. The equivalent circuit, used for fitting, is displayed in the upper left panel of Figure S1. They can be attributed to bulk and grain boundary resistance according to their capacitances.[24] The semicircle at higher frequencies (lower real impedance) corresponds to the bulk contribution.[24]

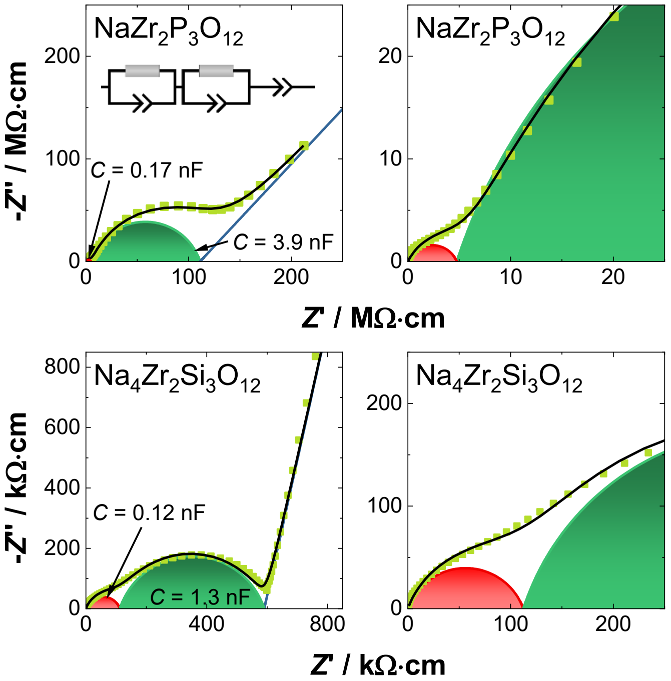

Figure S1. Impedance spectra of $NaZr_2P_3O_{12}$ and $Na_4Zr_2Si_3O_{12}$ measured at room temperature. The equivalent circuit used for fitting the impedance spectra is shown in the upper left panel. The right panels are close-up version of the left panels.

Subsequently, ionic bulk conductivities are calculated from the bulk resistances. $NaZr_2P_3O_{12}$ and $Na_4Zr_2Si_3O_{12}$ exhibit low bulk ionic conductivities of $2.1 \cdot 10^{-7}$ S·cm^{-1} and $8.8 \cdot 10^{-5}$ S·cm^{-1} at room temperature, and high activation energies, as displayed in Figure S2. In $NaZr_2P_3O_{12}$ the Na(2) site possess a high potential energy and is fully unoccupied. Therefore, ionic jumps (via the Na(3) site) to this lattice position face a

high activation barrier, resulting in a low ionic conductivity. The path onto another Na(1) site is long and involves multiple individual jumps (Na(1)-Na(3)-Na(2)-Na(3)-Na(1)). On the contrary, in $Na_4Zr_2Si_3O_{12}$ all Na(1) and Na(2) positions are occupied, so the diffusion path towards the next occupied site is shortened to Na(1)-Na(3)-Na(2), slightly increasing the ionic conductivity. However, the lack of partial site occupancy in $Na_4Zr_2Si_3O_{12}$, leads to a lack of vacancies for diffusion, explaining the low ionic conductivity.

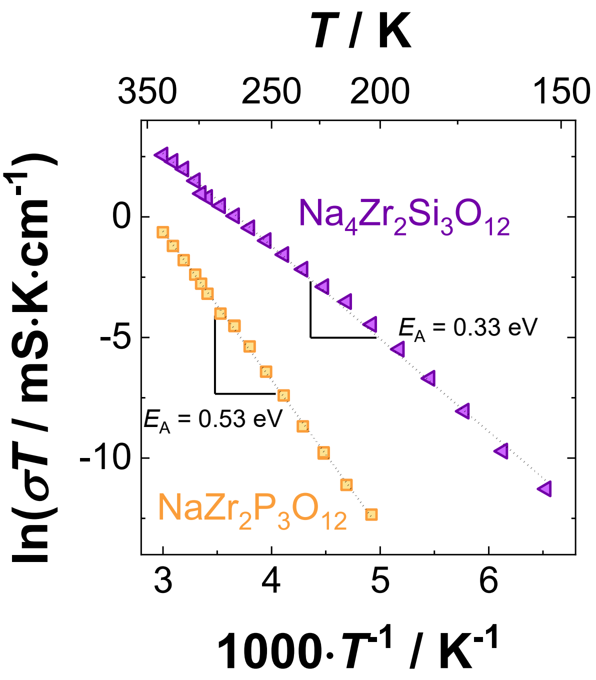

Figure S2. Arrhenius plot of the ionic bulk conductivities of $NaZr_2P_3O_{12}$ and $Na_4Zr_2Si_3O_{12}$ with corresponding activation energies.

Section S3: Rietveld refinements

Pawley fits and Rietveld refinements for phase analyses and structure analysis were carried out using TOPAS academics V7.21.[25] For each experiment the diffractogram acquired at room temperature was analyzed using a Pawley fit. Background, instrument parameters, peak shape, and lattice parameters obtained were subsequently used as starting parameter for the Rietveld refinements of the measurement performed at room temperature. Diffractograms at all other temperatures were refined sequentially, i.e., the refined parameters of the previous temperature step served as input for the subsequent one. Each profile was refined by (1) a scale factor, (2) ten free background coefficients of a Chebyshev polynomial function, (3) zero point offset, (4) peak shape parameters of a modified Thompson–Cox–Hastings pseudo-Voigt function[26], (5) a full axial model to account for the

asymmetry of the reflections caused by axial divergence[27], (6) the lattice parameters, (7) the general fractional atomic coordinates and finally (8) isotropic atomic displacement parameters. Each set of parameters was refined only once all previous parameters had converged by iterative refinement. Exemplary X-ray diffractograms with their corresponding Rietveld refinements are shown in Figure S3. Structural parameters at 150 K, room temperature, and the highest measured temperature of both components are given in Tables S4-S9. Due to the low atomic number and therefore low X-ray cross sections of oxygen, combined with low thermal displacements, both oxygen sites in $Na_4Zr_2Si_3O_{12}$ were assigned the same thermal displacement parameter.

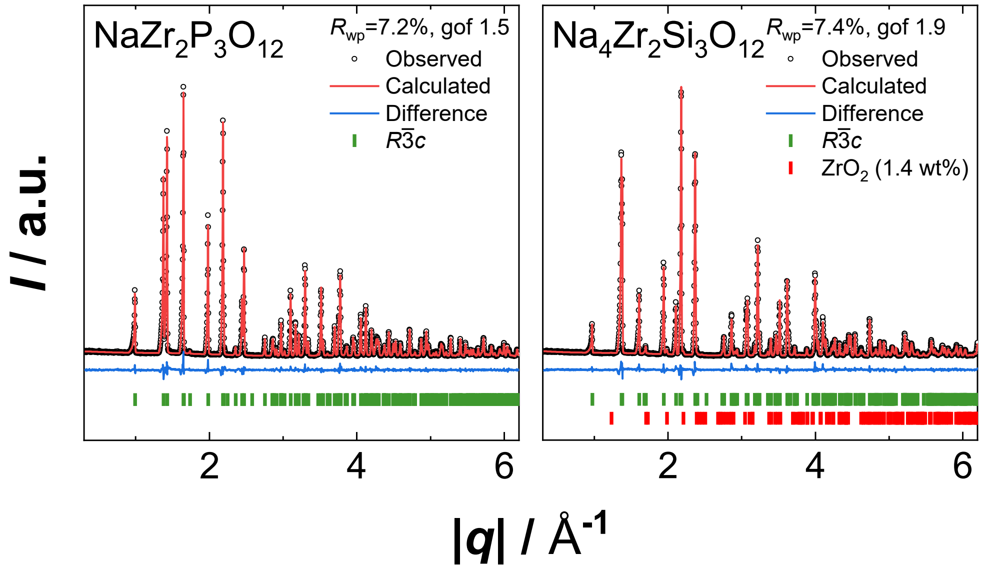

Figure S3. Exemplary Rietveld refinements acquired at 270 K of $NaZr_2P_3O_{12}$ and $Na_4Zr_2Si_3O_{12}$ with fit, difference curve, and reflection positions.

Table S4. Structure parameters of the refinement of $NaZr_2P_3O_{12}$ at 150 K.

**$NaZr_2P_3O_{12}$, 150 K**						
Lattice parameter: $a = b$ = 8.81237(16) Å; c = 22.7078(5) Å						
R_{wp} = 8.7%, GoF = 1.9						
Space group: $R\bar{3}c$ (No. 167) Impurity phase: -						
**Atom**	**Wyckoff position**	**Atomic coordinates**			**Occup.**	**B_{iso} / Å^2**
		x	***y***	***z***		
**Na(1)**	6b	0	0	0	1	1.26(14)

**Zr**	12c	0	0	0.14546(5)	1	0.15(3)
**P**	18e	0.2911(3)	0	0.25	1	0.49(7)
**O(1)**	36f	0.1930(4)	0.1692(5)	0.0865(2)	1	0.15(11)
**O(2)**	36f	0.0157(6)	0.2012(5)	0.1950(2)	1	0.22(11)

Table S5. Structure parameters of the refinement of $NaZr_2P_3O_{12}$ at room temperature (298 K).

$NaZr_2P_3O_{12}$, 298 K

Lattice parameter: $a = b = 8.8091(3)$ Å; $c = 22.7858(9)$ Å

R_{wp} = 6.9%, GoF = 3.0

Space group: $R\bar{3}c$ (No. 167) Impurity phase: -

**Atom**	**Wyckoff position**	**Atomic coordinates**			**Occup.**	B_{iso} / Å^2
		x	***y***	***z***		
**Na(1)**	6b	0	0	0	1	2.8(2)
**Zr**	12c	0	0	0.14559(5)	1	0.14(4)
**P**	18e	0.2907(3)	0	0.25	1	0.68(8)
**O(1)**	36f	0.1927(4)	0.1697(5)	0.0873(2)	1	0.36(11)
**O(2)**	36f	0.0176(6)	0.2014(5)	0.1952(2)	1	0.75(11)

Table S6. Structure parameters of the refinement of $NaZr_2P_3O_{12}$ at 623 K.

$NaZr_2P_3O_{12}$, 623 K

Lattice parameter: $a = b = 8.7973(3)$ Å; $c = 22.9636(10)$

R_{wp} = 7.6%, GoF = 3.3

Space group: $R\bar{3}c$ (No. 167) Impurity phase: -

**Atom**	**Wyckoff position**	**Atomic coordinates**			**Occup.**	B_{iso} / Å^2
		x	***y***	***z***		
**Na(1)**	6b	0	0	0	1	6.8(3)

**Zr**	12c	0	0	0.14617(5)	1	0.45(4)
**P**	18e	0.2891(4)	0	0.25	1	0.91(9)
**O(1)**	36f	0.1938(5)	0.1688(5)	0.0890(2)	1	1.24(14)
**O(2)**	36f	0.0220(6)	0.2029(6)	0.1962(2)	1	1.4(2)

Table S7. Structure parameters of the refinement of $Na_4Zr_2Si_3O_{12}$ at 150 K.

$Na_4Zr_2Si_3O_{12}$, 150 K

Lattice parameter: *a* = *b* = 9.19184(16) Å; *c* = 22.1163(5) Å

R_{wp} = 7.9%, *GoF* = 2.1

Space group: $R\bar{3}c$ (No. 167) Impurity phase: ZrO_2 (1.4 wt%)

**Atom**	**Wyckoff position**	**Atomic coordinates**			**Occup.**	B_{iso} **/ Å²**
		x	***y***	***z***		
**Na(1)**	6b	0	0	0	1	1.09(14)
**Na(2)**	18e	−0.3643(4)	0	1/4	1	1.66(11)
**Zr**	12c	0	0	0.14644(5)	1	0.19(3)
**Si**	18e	0.2971(3)	0	1/4	1	0.45(7)
**O(1)**	36f	0.1833(4)	0.1654(4)	0.0844(2)	1	0.24(7)
**O(2)**	36f	0.1868(5)	−0.0149(5)	0.1919(2)	1	0.24(7)

Table S8. Structure parameters of the refinement of $Na_4Zr_2Si_3O_{12}$ at room temperature (298 K).

$Na_4Zr_2Si_3O_{12}$, 298 K

Lattice parameter: *a* = *b* = 9.1773(2) Å; *c* = 22.1813(7) Å

R_{wp} = 4.6%, *GoF* = 2.3

Space group: $R\bar{3}c$ (No. 167) Impurity phase: ZrO_2 (1.1 wt%)

**Atom**	**Wyckoff position**	**Atomic coordinates**			**Occup.**	B_{iso} **/ Å²**
		x	***y***	***z***		

**Na(1)**	6b	0	0	0	1	1.9(2)
**Na(2)**	18e	−0.3629(4)	0	1/4	1	2.82(12)
**Zr**	12c	0	0	0.14676(4)	1	0.48(3)
**Si**	18e	0.2968(3)	0	1/4	1	0.54(7)
**O(1)**	36f	0.1858(3)	0.1648(4)	0.0860(2)	1	0.09(9)
**O(2)**	36f	0.1859(5)	−0.0152(5)	0.1918(2)	1	0.80(11)

Table S9. Structure parameters of the refinement of $Na_4Zr_2Si_3O_{12}$ at 618 K.

$Na_4Zr_2Si_3O_{12}$, 618 K
Lattice parameter: $a = b$ = 9.1737(2) Å; c = 22.3732(7) Å
R_{wp} = 4.7%, GoF = 2.3
Space group: $R\bar{3}c$ (No. 167) Impurity phase: ZrO_2 (1.1 wt%)

**Atom**	**Wyckoff position**	**Atomic coordinates**			**Occup.**	B_{iso} **/ Å²**
		x	***y***	***z***		
**Na(1)**	6b	0	0	0	1	3.1(2)
**Na(2)**	18e	−0.3584(5)	0	1/4	1	5.0(2)
**Zr**	12c	0	0	0.14726(5)	1	0.76(3)
**Si**	18e	0.2954(3)	0	1/4	1	0.87(7)
**O(1)**	36f	0.1873(4)	0.1635(5)	0.0872(2)	1	0.35(10)
**O(2)**	36f	0.1845(7)	−0.0174(6)	0.1931(2)	1	1.64(13)

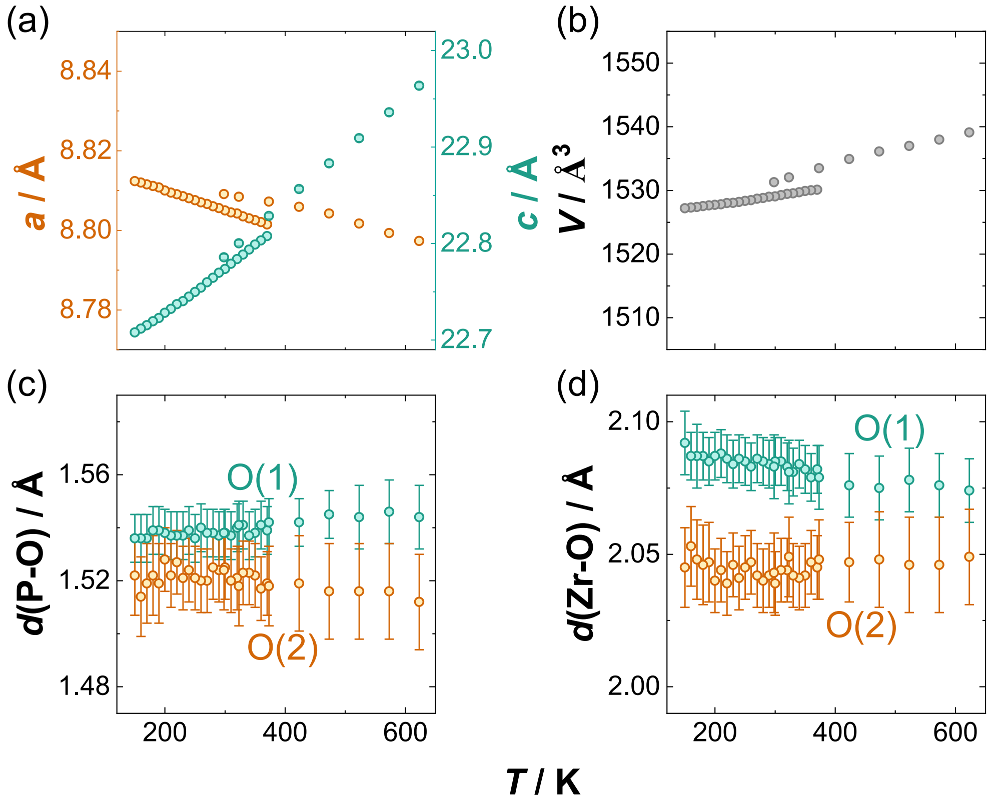

Figure S4. Analysis of the temperature-dependent structure of $NaZr_2P_3O_{12}$. (a) Unit cell parameters. (b) Unit cell volume in hexagonal representation. (c) Evolution of the P-O bond lengths with temperature. (d) Evolution of the Zr-O bond lengths with temperature.

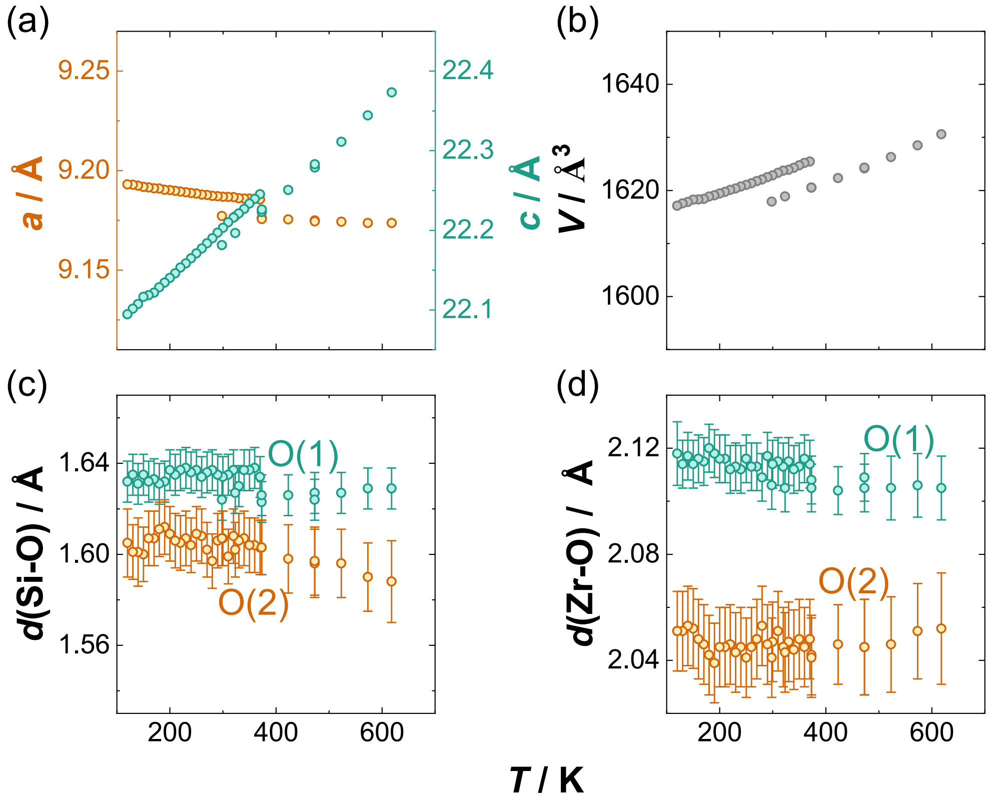

Figure S5. Analysis of the temperature-dependent structure of $Na_4Zr_2Si_3O_{12}$. (a) Unit cell parameters. (b) Unit cell volume in hexagonal representation. (c) Evolution of the Si-O bond lengths with temperature. (d) Evolution of the Zr-O bond lengths with temperature.

Analyzing the variations of rotational angles of the ZrO_6 and $(P/Si)O_4^{3/4-}$ polyhedra building up the framework requires a more detailed understanding of their degrees of freedom. The ZrO_6^{8-} octahedra are formed by two equilateral triangles, each aligned parallel to the *ab*-plane and composed of 3 O(1) or O(2) atoms, respectively. Although restricted in the *ab*-plane, they can rotate independently from each other around the *c*-axis. The O(1) and O(2) ions are offset to the *a*-axis (Figure S6a) by a small angle plus a integer multiple of 60° (i.e. an offset of 5° would mean the ion can be offset by 5°, 65°, 125°, … 305° to the axis). Both angles differ by less than 3° and their difference does not change by more than 2° throughout the entire temperature range for both compounds (Figure S6b). This reaffirms the nearly ideal octahedral shape, which would have no offset at all (0°), and the assumption of rigid units. Contrary, the rotation of the $(P/Si)O_4^{3/4-}$ tetrahedra is not constrained by symmetry. Due to the symmetry of the hexagonal lattice, the *a*- and *b*-axes are symmetrically identical. Therefore, only the rotation against the *a*-axis and the rotation against the *c*-axis are examined. Strictly speaking, the ions on the O(1) and O(2) sites need separate investigation, as they can

move independently from each other, forming different angles. However, because of the rigidity and their almost perfect tetrahedral shape (Figure S4c and Figure S5c) only the rotations of the O(2) site are discussed here. Again, for all investigated angles a slight increase of rotation angle is noticed with increasing temperature.

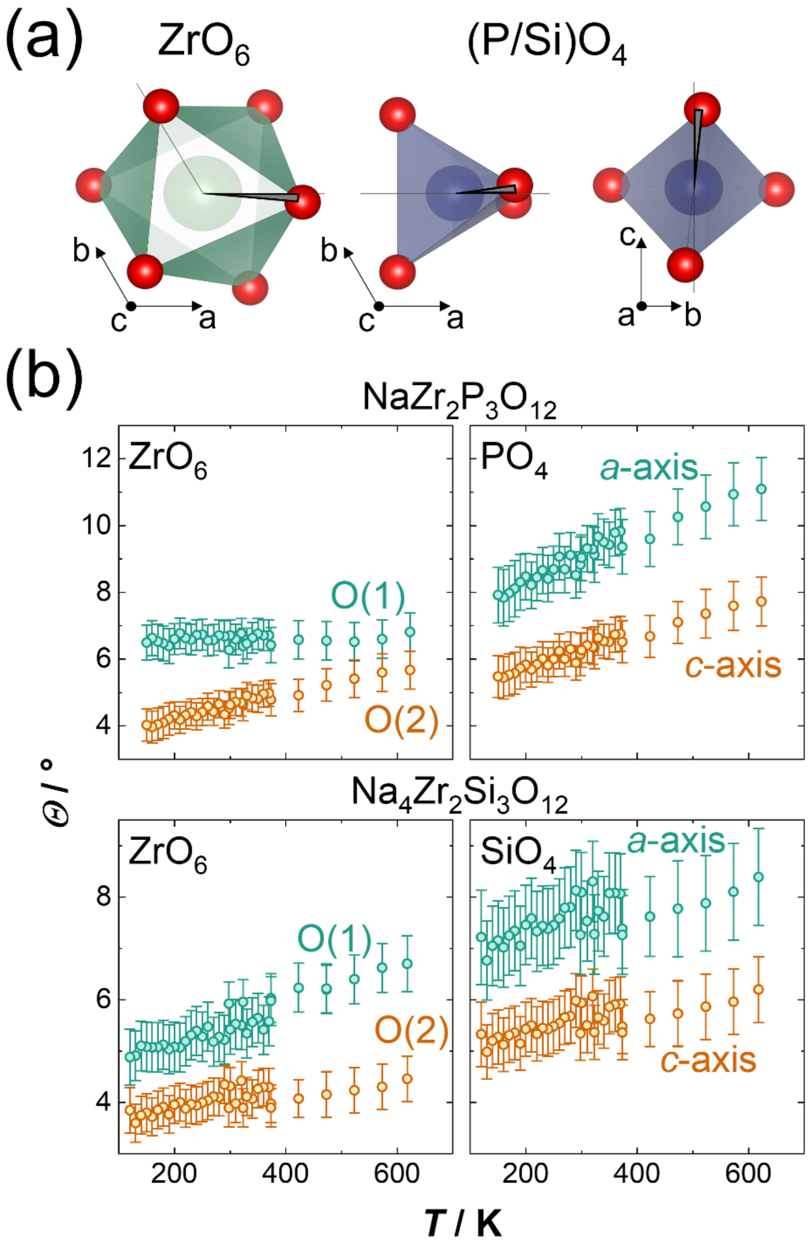

Figure S6. (a) Polyhedra illustrating the rotation angles (grey) of the ZrO_6^{8-} octahedra and the $(P/Si)O_4^{3/4-}$ tetrahedra against the unit cell axes. (b) Temperature dependence of the rotation of the $(P/Si)O_4^{3/4-}$ tetrahedra and ZrO_6^{8-} octahedra against the unit cell axes in both compounds.

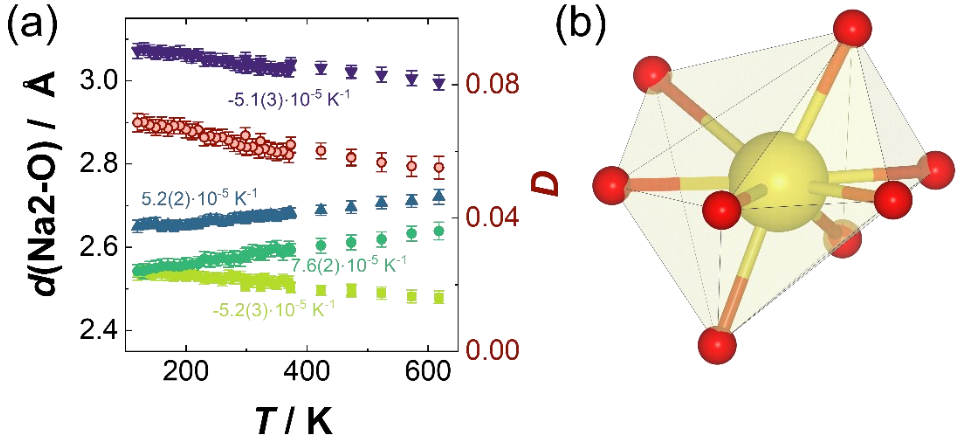

Figure S7. (a) Temperature-dependent Na(2)-O bond lengths in $Na_4Zr_2Si_3O_{12}$ with their respective expansion coefficients (left y-axis) and the distortion index of the coordination environment (right y-axis). The distortion is slightly decreasing with increasing temperature. (b) Na(2) polyhedron illustrating the distorted bonding environment with four distinct bond length, each occurring twice.

Section S4: Einstein oscillators

The Einstein model of a solid assumes that all ions vibrate at the same frequency. The hypothetical phonon density of states would be a delta function at this frequency. Despite this drastic simplification, the Einstein model is able to describe the vibrational frequencies of ions, especially the mobile species in solid electrolytes, with good accuracy. In first approximation, these loosely bound ions can be assumed to vibrate independently of their surroundings.[28] The Einstein frequency can be easily obtained using the slope of the isotropic thermal displacement parameter with temperature:

$$\frac{\mathrm{d}B_{\mathrm{iso}}}{\mathrm{d}T} = \frac{2k_{\mathrm{B}}}{m\nu_{\mathrm{E}}^{2}}$$
$$\Rightarrow \nu_E = \sqrt{\frac{2k_{\mathrm{B}}}{m}\cdot\frac{\mathrm{d}T}{\mathrm{d}B_{\mathrm{iso}}}} \tag{4}$$

with B_{iso} denoting the isotropic thermal displacement parameter, T the thermodynamic temperature, k_B the Boltzmann constant, m the mass of the ion (here sodium) and ν_E the Einstein frequency.

Section S5: Crystal Orbital Hamilton Populations

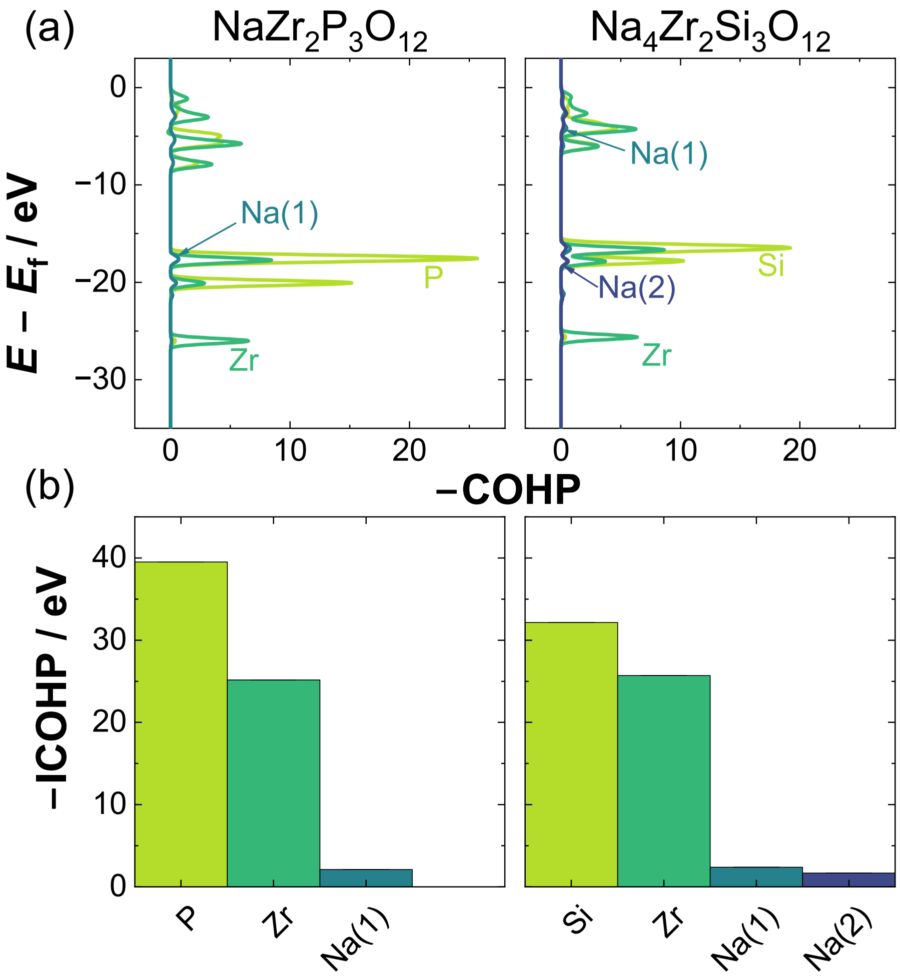

Figure S8. (a) Crystal Orbital Hamilton Populations (COHP) of the different cation sites in $NaZr_2P_3O_{12}$ and $Na_4Zr_2Si_3O_{12}$. No anti-bonding states (corresponding to a positive COHP) below the Fermi level are observed. (b) The integrated COHP (ICOHP) hints towards the bonding strength of the cations. The sodium ions clearly have the lowest bonding strength.

Section S6: Phonon band structure and phonon DOS

Average phonon frequencies used in the discussion of the phonon density of states are calculated by weighing the frequencies *v* by the phonon density of states *g*(*v*):

$$\nu_{\mathrm{avg}} = \frac{\sum_i g(\nu_i) \cdot \nu_i}{\sum_i \nu_i} \tag{5}$$

To investigate the jump-direction-projected DOS, the vector from the relaxated Na(1) or Na(2) site to the intermediate Na(3) site was calculated. This vector was then taken as direction to project the phonon DOS using the phonopy python package.

The spatial distribution of average vibrational frequencies (Figures 8a and 8b in the main text) actually comprises of a multitude of faces. To construct this spherical polyhedron a mesh of points was mapped onto a sphere ensuring uniform density and the phonon DOS for each point was calculated using each point's vector to the sphere's center as direction for the projection. Afterwards a spherical Voronoi diagram based on these points were constructed and each Voronoi region color coded according to the average frequency of the phonon DOS projected in the direction of its center point. Finally, the neighboring sites were added for visual reference of the orientation of each sphere.

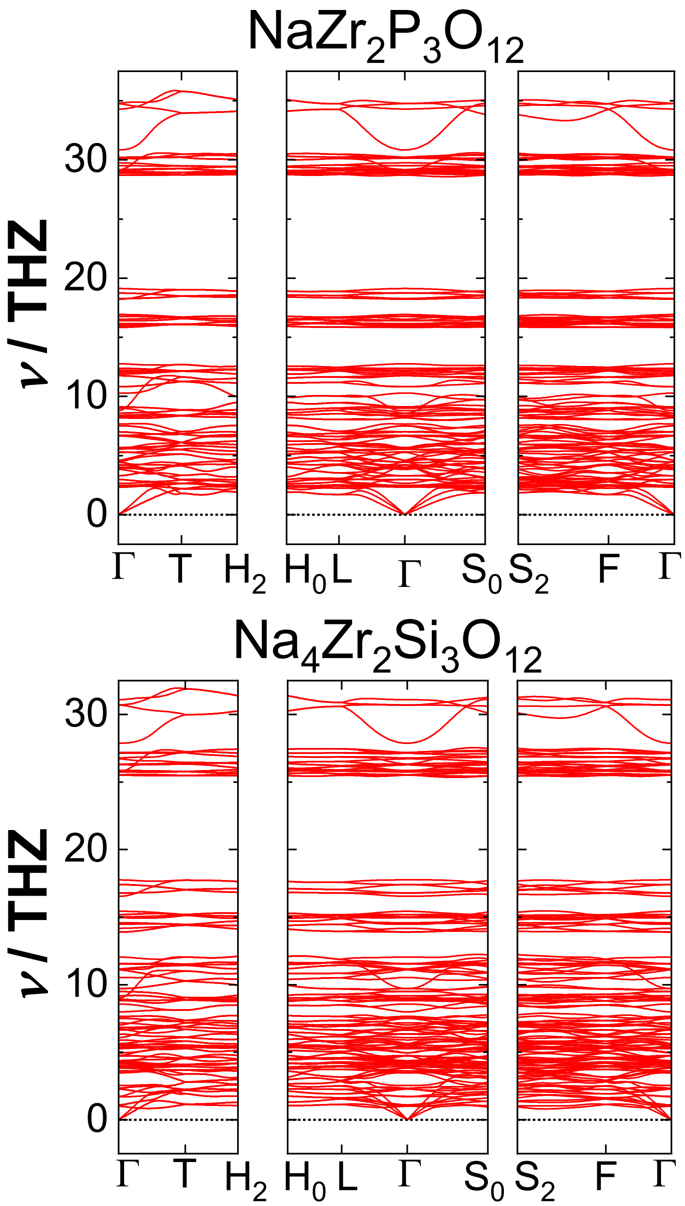

Figure S9. Full phonon band structure of $NaZr_2P_3O_{12}$ and $Na_4Zr_2Si_3O_{12}$. With a few exemptions all optical modes are almost dispersionless.

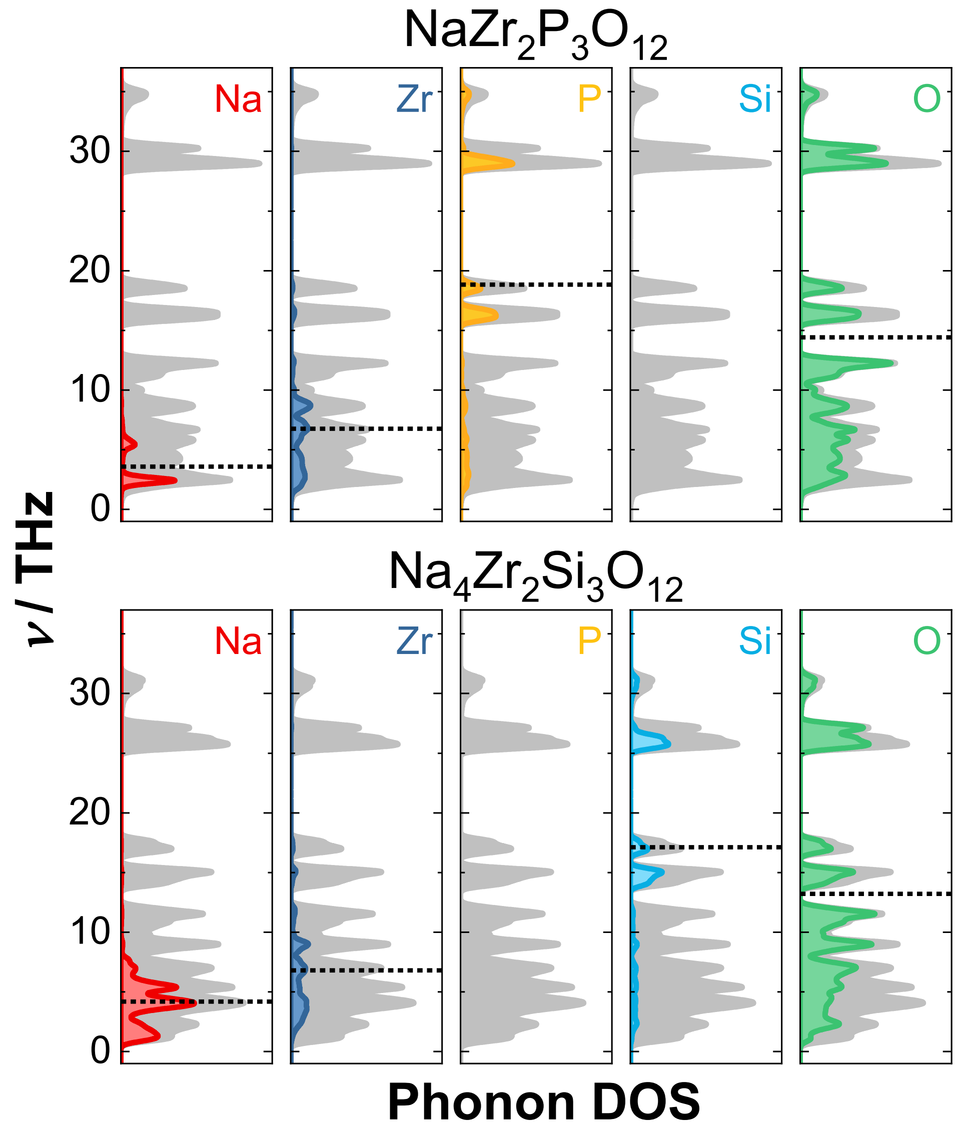

Figure S10. Atom projected phonon density of states (DOS) of $NaZr_2P_3O_{12}$, and $Na_4Zr_2Si_3O_{12}$. Dashed lines represent the average phonon frequency of that respective ion.

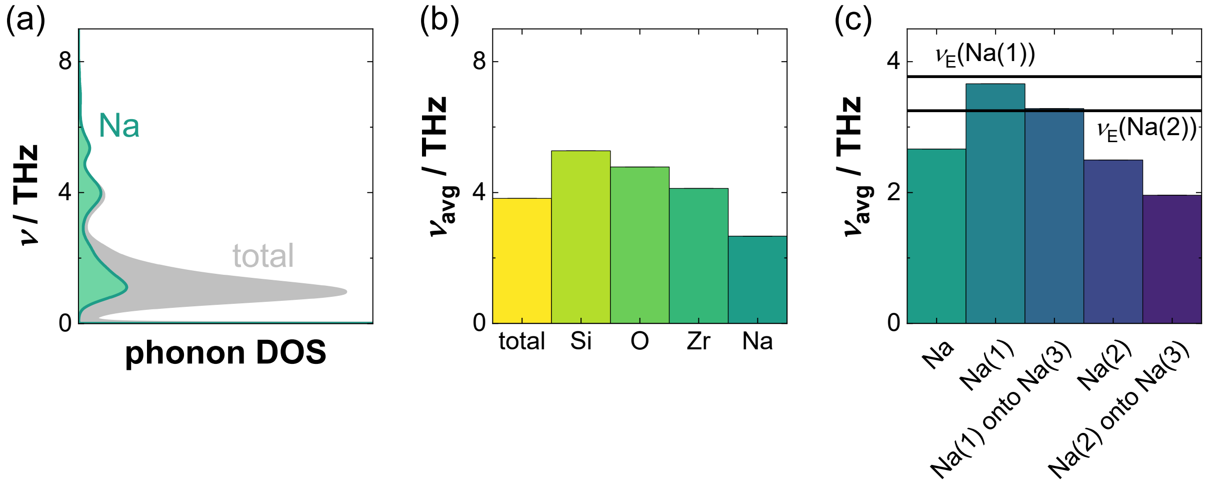

Figure S11. (a) Total (grey) and sodium-projected (turquoise) phonon occupation weighed density of states (DOS) of $Na_4Zr_2Si_3O_{12}$. The phonon DOS for higher frequencies is too small to be shown. (b) Phonon occupation-weighted average frequencies of the total and atom-projected DOS in $Na_4Zr_2Si_3O_{12}$. (c) Phonon occupation weighed average frequency of site- and jump direction-projected DOS in $Na_4Zr_2Si_3O_{12}$. Experimentally found Einstein frequencies are given by horizontal lines. The temperature for the phonon occupation was set to 300 K in all cases.

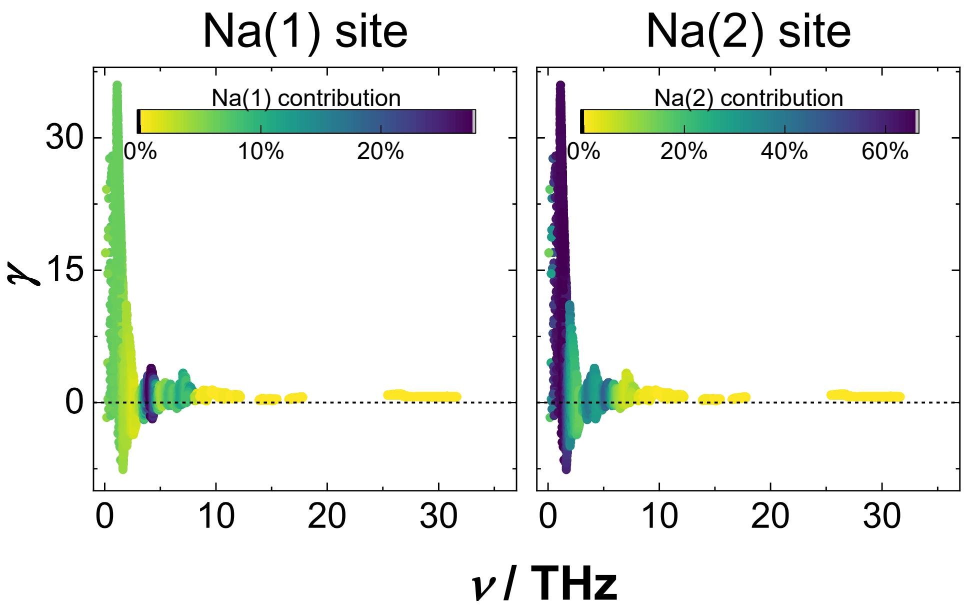

Figure S12. Grüneisen parameter of $Na_4Zr_2Si_3O_{12}$. The ratio of site-projected phonon DOS of the Na(1) (left panel) and Na(2) (right panel) to total phonon DOS, i.e., contribution of the respective sodium ions to vibrations at a given frequency, is colour-coded.

Section S7: Electronic contribution to the thermal conductivity

The total thermal conductivity κ_{tot} is the sum of the lattice thermal conductivity κ_{lat}, discussed in the manuscript, and the contribution of heat carried by mobile electrons κ_{el}. While latter is the main source of thermal conduction in metals, it can be usually neglected in electronic insulators like solid electrolytes. However, given the low lattice thermal conductivity, the electronic thermal conductivity is estimated here. This can be done using the Wiedeman-Franz law and literature values[29] for the electronic conductivity σ_{el} of NASICON electrolytes.

$$\kappa_{\mathrm{el}} = \sigma_{\mathrm{el}} \cdot L \cdot T = 1 \cdot 10^{-6} \frac{\mathrm{S}}{\mathrm{m}} \cdot 2.44 \cdot 10^{-8} \frac{\mathrm{V}^2}{\mathrm{K}^2} \cdot 300\ \mathrm{K} = 7 \cdot 10^{-12} \frac{\mathrm{W}}{\mathrm{mK}} \quad (6)$$

L is the Lorenz number and T the temperature. At room temperature the contribution of electrons to the total thermal conductivity is therefore more than 11 orders of magnitude smaller than the lattice thermal conductivity and can be therefore neglected.

Section S8: Scanning electron microscopy

In order to investigate the interior of the pellets, they were broken in half and their cross-section was examined by scanning electron microscopy. The samples were sputter-coated with gold and fixed to the sample holder with double-sided carbon tape. A Carl Zeiss AURIGA CrossBeam working station (accelerating voltage of 3 kV) equipped with an In-lens detector was used for characterization. In both compounds secondary particles with sizes mostly between 1 µm and 5 µm were found. However, these particles themselves are microstructured (Figure S13*Figure S13*), leaving the (distribution of) crystallite sizes unknown. Energy-dispersive X-ray spectroscopy (EDX) showed a homogenous distribution of all elements. As the information depth of EDX is in the range of 1 µm, the small particles of <100 nm in size seen for high magnifications of $Na_4Zr_2Si_3O_{12}$ (Figure S13d) cannot be identified with this technique. One possibility is that these particles are residues of the TiO_2 nano powder used as sintering aid. However, they could also be ZrO_2 side phase or small $Na_4Zr_2Si_3O_{12}$ particles. As their volume is very small and their density on the surface of the larger particles also rather sparse, they will not affect the thermal conductivity, which is a bulk conductivity or contribute noticeably to thermal boundary resistance.

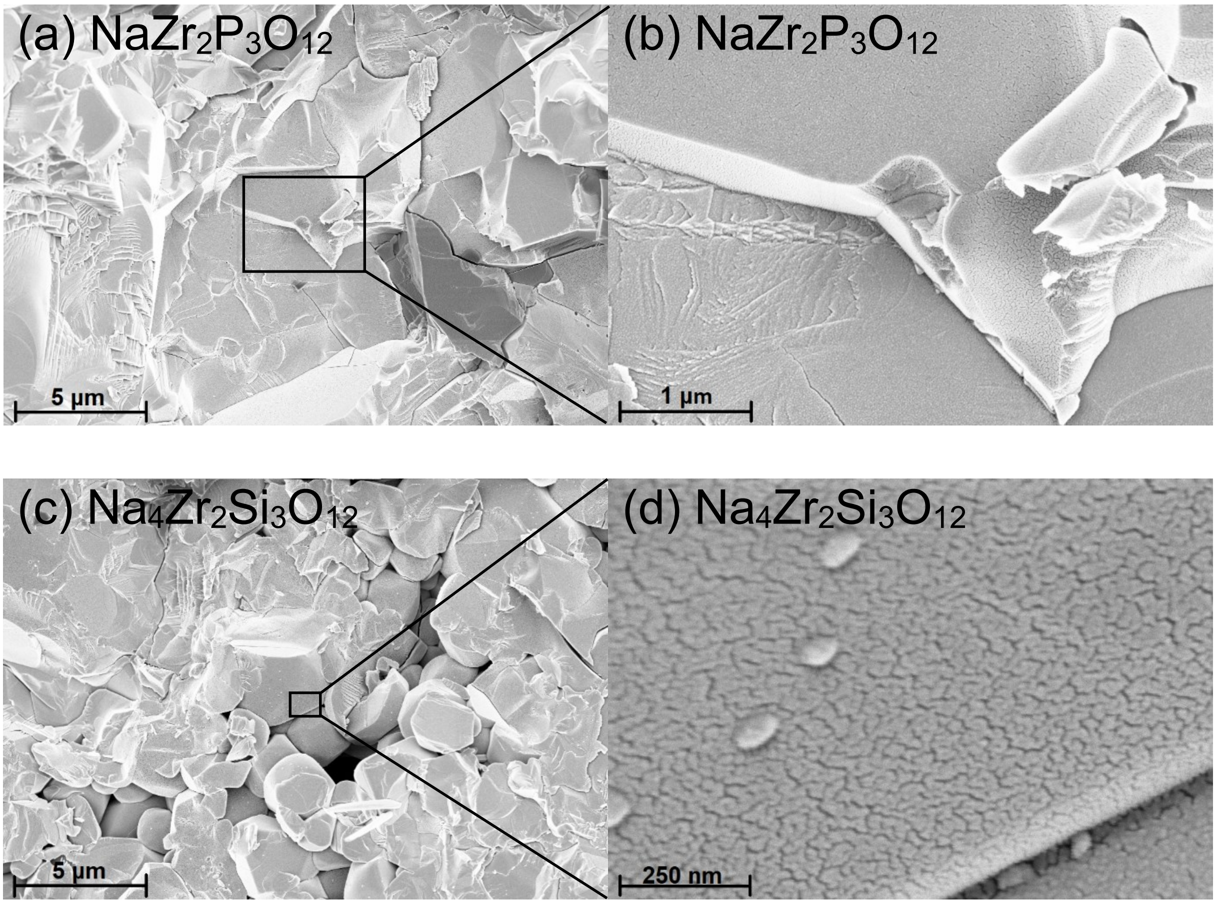

Figure S13. Scanning electron microscopy images of $NaZr_2P_3O_{12}$ (a) and (b) and $Na_4Zr_2Si_3O_{12}$ (c) and (d). In both compounds particles with a microstructure were observed. The boxes in (a) and (c) highlight that panels (b) and (d) are zoomed in versions. The sizes of the particles represent upper limits for the crystallite sizes assumed in the modelling of the thermal conductivity.

References

(1) Kamali, K.; Ravindran, T. R.; Ravi, C.; Sorb, Y.; Subramanian, N.; Arora, A. K. Anharmonic phonons of NaZr2(PO4)3 studied by Raman spectroscopy, first-principles calculations, and x-ray diffraction. *Phys. Rev. B* **2012**, *86* (14), 144301. DOI: 10.1103/PhysRevB.86.144301.

(2) Sukhanov, M. V.; Pet'kov, V. I.; Firsov, D. V. Sintering mechanism for high-density NZP ceramics. *Inorg. Mater.* **2011**, *47* (6), 674–678. DOI: 10.1134/S0020168511060197.

(3) Takeuchi, T.; Bétourné, E.; Sinclair, D. C.; Tabuchi, M.; West, A. R.; Kageyama, H. Densification and conductivity enhancement of Na4Zr2Si3O12-based solid

electrolytes using TiO2 as a sintering aid. *Solid State Ionics* **1999**, *120* (1-4), 33–41. DOI: 10.1016/S0167-2738(99)00004-1.

(4) Wang, X.; Liu, Z.; Tang, Y.; Chen, J.; Wang, D.; Mao, Z. Low temperature and rapid microwave sintering of Na3Zr2Si2PO12 solid electrolytes for Na-Ion batteries. *J. Power Sources* **2021**, *481*, 228924. DOI: 10.1016/j.jpowsour.2020.228924.

(5) Cape, J. A.; Lehman, G. W. Temperature and Finite Pulse-Time Effects in the Flash Method for Measuring Thermal Diffusivity. *J. Appl. Phys.* **1963**, *34* (7), 1909–1913. DOI: 10.1063/1.1729711.

(6) Blumm, J.; Opfermann, J. Improvement of the mathematical modeling of flash measurements. *High Temp.-High Press.* **2002**, *34* (5), 515–521. DOI: 10.1068/htjr061.

(7) Pet'kov, V. I.; Orlova, A. I.; Kazantsev, G. N.; Samoilov, S. G.; Spiridonova, M. L. Thermal Expansion in the Zr and 1-, 2-Valent Complex Phosphates of NaZr2(PO4)3 (NZP) Structure. *J. Therm. Anal. Calorim.* **2001**, *66* (2), 623–632. DOI: 10.1023/A:1013145807987.

(8) Oota, T.; Yamai, I. Thermal Expansion Behavior of NaZr2(PO4)3Type Compounds. *J. Am. Ceram. Soc.* **1986**, *69* (1), 1–6. DOI: 10.1111/j.1151-2916.1986.tb04682.x.

(9) Kresse; Furthmüller. Efficient iterative schemes for ab initio total-energy calculations using a plane-wave basis set. *Phys. Rev. B: Condens. Matter* **1996**, *54* (16), 11169–11186. DOI: 10.1103/physrevb.54.11169.

(10) Kresse, G.; Furthmüller, J. Efficiency of ab-initio total energy calculations for metals and semiconductors using a plane-wave basis set. *Comput. Mater. Sci.* **1996**, *6* (1), 15–50. DOI: 10.1016/0927-0256(96)00008-0.

(11) Kresse, G.; Joubert, D. From ultrasoft pseudopotentials to the projector augmented-wave method. *Phys. Rev. B* **1999**, *59* (3), 1758–1775. DOI: 10.1103/PhysRevB.59.1758.

(12) Blöchl, P. E. Projector augmented-wave method. *Phys. Rev. B: Condens. Matter* **1994**, *50* (24), 17953–17979. DOI: 10.1103/PhysRevB.50.17953.

(13) Perdew, J. P.; Burke, K.; Ernzerhof, M. Generalized Gradient Approximation Made Simple. *Phys. Rev. Lett.* **1996**, *77* (18), 3865–3868. DOI: 10.1103/PhysRevLett.77.3865.

(14) Togo, A.; Chaput, L.; Tanaka, I. Distributions of phonon lifetimes in Brillouin zones. *Phys. Rev. B* **2015**, *91* (9). DOI: 10.1103/PhysRevB.91.094306.

(15) Togo, A.; Tanaka, I. First principles phonon calculations in materials science. *Scr. Mater.* **2015**, *108*, 1–5. DOI: 10.1016/j.scriptamat.2015.07.021.
(16) Togo, A. First-principles Phonon Calculations with Phonopy and Phono3py. *J. Phys. Soc. Jpn.* **2023**, *92* (1). DOI: 10.7566/JPSJ.92.012001.
(17) Simoncelli, M.; Marzari, N.; Mauri, F. Unified theory of thermal transport in crystals and glasses. *Nat. Phys.* **2019**, *15* (8), 809–813. DOI: 10.1038/s41567-019-0520-x.
(18) Simoncelli, M.; Marzari, N.; Mauri, F. Wigner Formulation of Thermal Transport in Solids. *Phys. Rev. X* **2022**, *12* (4). DOI: 10.1103/PhysRevX.12.041011.
(19) Gonze, X.; Charlier, J.; Allan, D. C.; Teter, M. P. Interatomic force constants from first principles: The case of alpha -quartz. *Phys. Rev. B: Condens. Matter* **1994**, *50* (17), 13035–13038. DOI: 10.1103/PhysRevB.50.13035.
(20) Gonze, X.; Lee, C. Dynamical matrices, Born effective charges, dielectric permittivity tensors, and interatomic force constants from density-functional perturbation theory. *Phys. Rev. B* **1997**, *55* (16), 10355–10368. DOI: 10.1103/PhysRevB.55.10355.
(21) Dronskowski, R.; Bloechl, P. E. Crystal orbital Hamilton populations (COHP): energy-resolved visualization of chemical bonding in solids based on density-functional calculations. *J. Phys. Chem.* **1993**, *97* (33), 8617–8624. DOI: 10.1021/j100135a014.
(22) Maintz, S.; Deringer, V. L.; Tchougréeff, A. L.; Dronskowski, R. LOBSTER: A tool to extract chemical bonding from plane‐wave based DFT. *J. Comput. Chem.* **2016**, *37* (11), 1030–1035. DOI: 10.1002/jcc.24300.
(23) Naik, A. A.; Ueltzen, K.; Ertural, C.; Jackson, A. J.; George, J. LobsterPy: A package to automatically analyze LOBSTER runs. *JOSS* **2024**, *9* (94), 6286. DOI: 10.21105/joss.06286.
(24) Irvine, J. T. S.; Sinclair, D. C.; West, A. R. Electroceramics: Characterization by Impedance Spectroscopy. *Adv. Mater.* **1990**, *2* (3), 132–138. DOI: 10.1002/adma.19900020304.
(25) Coelho, A. A. TOPAS and TOPAS-Academic: an optimization program integrating computer algebra and crystallographic objects written in C++. *J. Appl. Crystallogr. (Journal of Applied Crystallography)* **2018**, *51* (1), 210–218. DOI: 10.1107/S1600576718000183.

(26) Thompson, P.; Cox, D. E.; Hastings, J. B. Rietveld refinement of Debye–Scherrer synchrotron X-ray data from Al 2 O 3. *J Appl Crystallogr* **1987**, *20* (2), 79–83. DOI: 10.1107/S0021889887087090.
(27) Cheary, R. W.; Coelho, A. A. An experimental investigation of the effects of axial divergence on diffraction line profiles. *Powder Diffr.* **1998**, *13* (2), 100–106. DOI: 10.1017/S0885715600009933.
(28) Bernges, T.; Hanus, R.; Wankmiller, B.; Imasato, K.; Lin, S.; Ghidiu, M.; Gerlitz, M.; Peterlechner, M.; Graham, S.; Hautier, G.; Pei, Y.; Hansen, M. R.; Wilde, G.; Snyder, G. J.; George, J.; Agne, M. T.; Zeier, W. G. Considering the Role of Ion Transport in Diffuson-Dominated Thermal Conductivity. *Adv. Energy Mater.* **2022**, *12* (22), 2200717. DOI: 10.1002/aenm.202200717.
(29) Gorai, P.; Famprikis, T.; Singh, B.; Stevanović, V.; Canepa, P. Devil is in the Defects: Electronic Conductivity in Solid Electrolytes. *Chem. Mater.* **2021**, *33* (18), 7484–7498. DOI: 10.1021/acs.chemmater.1c02345.
(30) Agne, M. T.; Voorhees, P. W.; Snyder, G. J. Phase Transformation Contributions to Heat Capacity and Impact on Thermal Diffusivity, Thermal Conductivity, and Thermoelectric Performance. *Adv. Mater.* **2019**, *31* (35), e1902980. DOI: 10.1002/adma.201902980. Published Online: Jul. 3, 2019.
(31) Rohde, M.; Mohsin, I. U. I.; Ziebert, C.; Seifert, H. J. Ionic and Thermal Transport in Na-Ion-Conducting Ceramic Electrolytes. *Int. J. Thermophys.* **2021**, *42* (10), 136. DOI: 10.1007/s10765-021-02886-x.
(32) Krenzer, G.; Kim, C.-E.; Tolborg, K.; Morgan, B. J.; Walsh, A. Anharmonic lattice dynamics of superionic lithium nitride. *J. Mater. Chem. A* **2022**, *10* (5), 2295–2304. DOI: 10.1039/D1TA07631K.
